# Synthesis-driven reverse metabolomics reveals 3-hydroxy *N*-acyl amides as gut microbial molecules

**DOI:** 10.1128/msystems.00839-26

**Published:** 2026-09-24

**Authors:** Victoria Deleray, Vincent Charron-Lamoureux, Kyle Vittali, Helena Mannochio-Russo, Crystal X. Wang, Corinn Walker, Karsten Zengler, Dionicio Siegel, Pieter C. Dorrestein

**Affiliations:** 1 Skaggs School of Pharmacy and Pharmaceutical Sciences, University of California San Diego 8784, La Jolla, California, USA; 2 Collaborative Mass Spectrometry Innovation Center, University of California San Diego 8784, La Jolla, California, USA; 3 Department of Chemistry and Biochemistry, University of California San Diego, Gilman Drive 8784, La Jolla, California, USA; 4 Department of Psychiatry, University of California San Diego, Gilman Drive 8784, La Jolla, California, USA; 5 Department of Pediatrics, University of California, San Diego 8784 , La Jolla, California, USA; 6 Department of Bioengineering, University of California, San Diego 8784 , La Jolla, California, USA; 7 Center for Microbiome Innovation, University of California, San Diego 8784 , La Jolla, California, USA; 8 Program in Materials Science and Engineering, University of California, San Diego, Gilman Drive 8784 , La Jolla, California, USA; 9 Soil Health Center, Scripps Institution of Oceanography, University of California San Diego, Gilman Drive 8784, La Jolla, California, USA; Pacific Northwest National Laboratory, Richland, Washington, USA; Colorado State University, Fort Collins, Colorado, USA

**Keywords:** microbial metabolism, reverse metabolomics, mass spectrometry, diabetes, ion mobility

## Abstract

3-Hydroxy *N*-acyl amides are bioactive lipids with reported anti-obesity and glucose-regulating effects, yet they are rarely detected in untargeted metabolomics studies because they are largely absent from existing spectral reference libraries. To address this gap, we synthesized an MS/MS spectral resource comprising 436 structurally diverse 3-hydroxy *N*-acyl amides, spanning 3- to 18-carbon chains with a wide range of amine headgroups, such as ornithine, valine, and dopamine. Using a synthesis-driven reverse metabolomics approach, we found 161,626 spectral matches across 54,744 publicly available files in untargeted metabolomics data sets, revealing widespread occurrences in biological samples, including human-derived specimens. Of these molecules detected through MS/MS spectral matching, 334 represent newly reported biological entities. We further confirmed their presence in human saliva, stool, and skin using retention time and ion mobility measurements. Frequent detection in microbial data sets and validation in communities of human-derived gut bacteria support microbial production. Several metabolites also showed altered abundance in individuals with diabetes mellitus, showing that this lipid class is modulated in human metabolic disease. Together, these findings establish 3-hydroxy *N*-acyl amides as a distinct and biologically relevant lipid class, and the accompanying MS/MS spectral resource will enable their broader recognition and study in untargeted metabolomics data.

**IMPORTANCE:**

Microbe-host interactions lead to the production of metabolites that play an important role in human health and disease, though the annotation of such metabolites remains scarce. We created a re-usable MS/MS spectral library of an underrepresented class of compounds—3-hydroxy *N*-acyl amides. Our study characterized the presence of over 400 3-hydroxy *N*-acyl amides among thousands of public untargeted metabolomics datasets. We found over 100,000 instances of these metabolites within microbial, plant, and animal data, and used orthogonal validation methods to confirm nine previously unreported molecules within human feces, saliva, and skin samples. We cultured communities of human-derived gut bacteria and found evidence of bacterial production of four 3-hydroxy *N*-acyl amides. Our results suggest that these metabolites play important roles in human metabolism and disease, and sharing this curated resource enables the detection of these metabolites in future studies.

## INTRODUCTION

The discovery of microbially derived and microbe-associated metabolites has transformed our understanding of host-microbe symbiosis, disease pathogenesis, bacterial virulence, and drug discovery (1–3). This discovery is due in large part to untargeted metabolomics, which enables a broad chemical readout of biological samples. Over the past decade, thousands of these studies have been deposited in public repositories, creating an opportunity to retrospectively analyze samples across diverse study designs and experimental conditions. However, only 8.1% of the mass spectrometry (MS)/MS spectra, which exist in this public data domain, have an MS/MS match to existing GNPS libraries, strongly limiting our ability to understand the breadth of structural diversity of metabolites and their biological roles (4).

Many mass spectrometry-based strategies have been developed to address this annotation gap, including synthesis-based reverse metabolomics. In traditional mass spectrometry-based metabolomics, experiments are designed prospectively, and metabolites that are associated with a phenotype are identified through various structure elucidation techniques, including MS/MS matching to known standards. Comparatively, reverse metabolomics starts with an MS/MS spectra that is searched across untargeted metabolomics repositories, such as GNPS/MassIVE, Metabolights, Metabolomics Workbench, and NORMAN, to discover phenotypic associations among various studies. The reverse metabolomics approach expands searchable chemical space to thousands of independent studies to enable the retrospective discovery of metabolites that are absent in current libraries, but detected in samples. Previously, this methodology enabled the discovery of hundreds of new bile amidates and drug conjugates, revealing potential mechanisms in the host-microbe interaction of irritable bowel disease, and treatment response (5–7).

**Fig 1:**
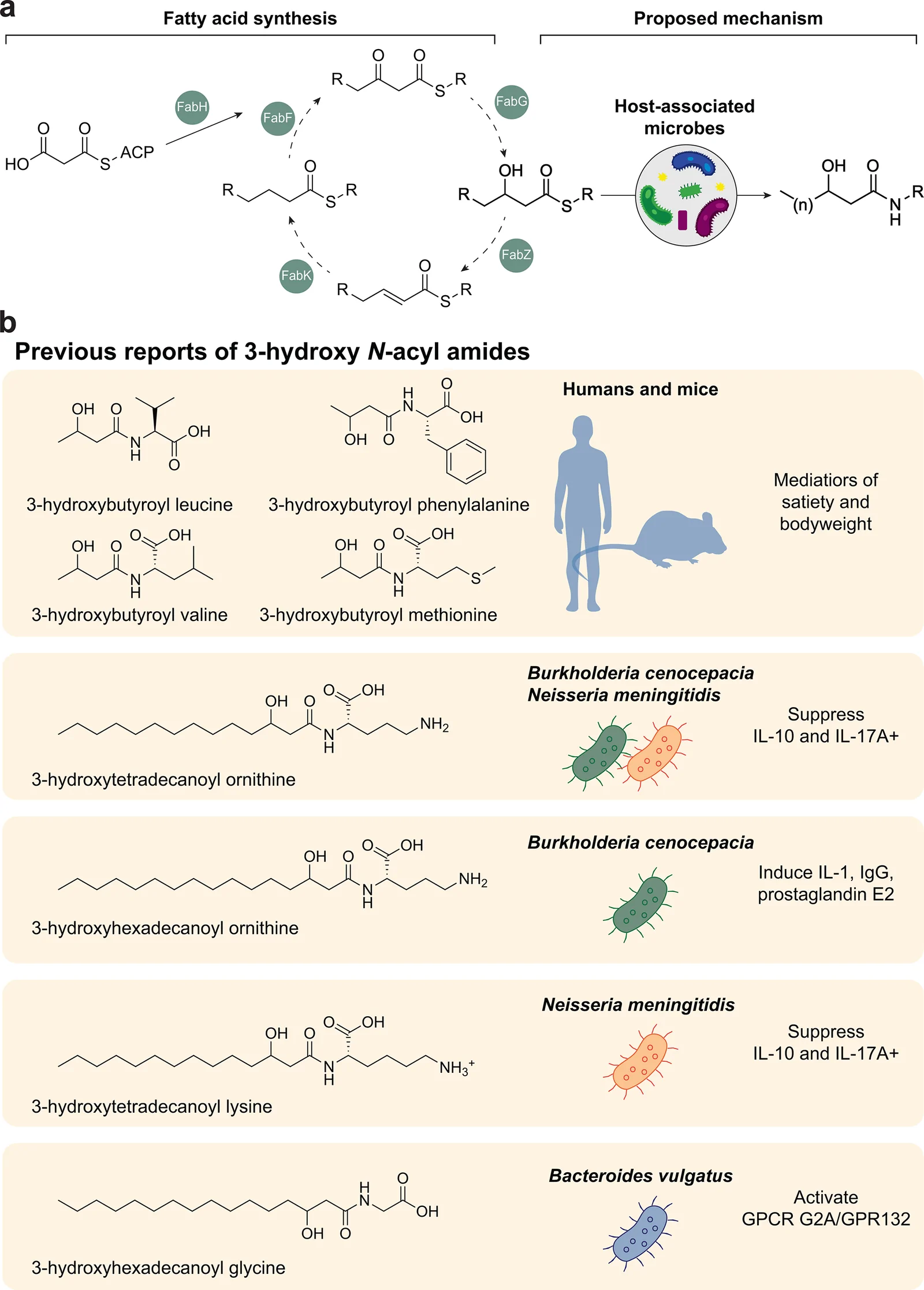
Production and functions of 3-hydroxy *N*-acyl amides. (**a**) Summary of known metabolic pathway of the synthesis of fatty acids to 3-hydroxy-fatty acyls occurring in the mitochondria (14). Our proposed mechanism shows the 3-hydroxy fatty acyl undergoing alternative metabolism to become a 3-hydroxy *N*-acyl amide. (**b**) Summary of previous reports of 3-hydroxy *N*-acyl amides, including structure, name, and organism it is found in, and known activities of these molecules (*in vivo* or *in vitro*) are referenced in the introduction.

Here, we have identified 3-hydroxy *N*-acyl amides as a poorly characterized class of microbial metabolites consisting of an amine head and β-hydroxylated fatty acid tail (Fig. 1a). Despite the limited reports of these structures, they have important functional roles in the virulence of pathogenic bacteria, host immune signaling, and mammalian energy metabolism (8–13) (Fig. 1b).

For example, studies that have aimed to identify virulence factors in pathogenic bacteria have singly isolated 3-hydroxytetradecanoyl ornithine and 3-hydroxytetradecanoyl lysine from *Neisseria meningitidis* (10)*,* the pathogen involved in meningitis and septicemia (10), and 3-hydroxyhexadecanoyl ornithine from *Burkholderia cenocepacia*, a bacteria involved in chronic cystic fibrosis infections (8). Following elucidation, these metabolites were described to stimulate various human immune pathways, highlighting their important role in the pathogenesis of bacterial infectious diseases (9, 11). A metagenome mining approach later revealed 3-hydroxyhexadecanoyl glycine as a bacterial metabolite with potential roles in autoimmune and metabolic inflammatory diseases (12). Although these metabolites were detected in bacterial and bovine medium sources, they have not been reported in humans. More recently, four β-hydroxybutyryl amino acid conjugates were identified as mammalian endogenous products of ketogenesis that regulate weight and satiety, though their potential role in host-microbe signaling has yet to be described (13). Together, these findings indicate that 3-hydroxy *N*-acyl amides have wide functions across bacterial and mammalian systems, yet their structural diversity and distribution across various biological systems remain largely unknown.

We hypothesized that synthesis-based reverse metabolomics would expand the known chemical space of 3-hydroxy *N*-acyl amides by generating a spectral library of synthetic standards that could be searched across public metabolomics data and make the molecules discoverable in future data sets through spectral library matching. Using this strategy, we mapped their occurrence across human, microbial, and plant samples by annotating tens of thousands of previously unannotated MS/MS spectra. Because an MS/MS match is a structural hypothesis, we performed MS/MS, retention time, and ion mobility matching against their matches in biological samples to provide further support for their annotation (this is at the level of the metabolomics standards initiative level 1 [15]). Nine of the previously unreported 3-hydroxy *N*-acyl amides were matched within various human tissue/biofluids, including feces, saliva, plasma, serum, skin, and bacterial cultures.

## RESULTS AND DISCUSSION

### Curation of the spectral library and pan-repository analysis

At present, the average annotation rate of all MS/MS using publicly accessible reference libraries in the major repositories Metabolights, Metabolomics Workbench, and GNPS/MassIVE is roughly 8% (7). This annotation gap is particularly limited for metabolite classes, such as 3-hydroxy *N*-acyl amides, for which a few individually characterized molecules have demonstrated important biological roles, but no systemic reference spectral library exists. Their modular composition of diverse 3-hydroxy fatty acid tails conjugated to various amines suggests that the few reported structures represent only a fraction of the chemical diversity that exists for these molecules. To facilitate their annotation, we aimed to create a reference library by performing 55 multiplexed reactions containing mixtures of 19 3-hydroxy fatty acids and 42 amine head groups (Table S1). Each multiplexed reaction had one to three 3-hydroxy lipids and was reacted with four to 24 different amines to generate their corresponding amides. The head groups included proteinogenic amino acids (e.g., valine, tryptophan, and alanine), polyamines (e.g., putrescine, cadaverine, and ornithine), and amino acid metabolites (e.g., citrulline, serotonin, and dopamine). The lipid tails were chosen based on commercial availability and reasonable costs, and amines were chosen based on availability in the Dorrestein/Siegel laboratories. Theoretically, these reactions could yield 798 distinct 3-hydroxy *N*-acyl amides, of which 536 were detected by liquid chromatography tandem mass spectrometry (LC-MS/MS). Of the 262 non-detected products, this included 247 non-saturated hydroxy fatty acids. This could be due to several reasons, including inefficient electrospray ionization or instability of the starting fatty acid material in the given reaction conditions. Additionally, the multiplexed synthesis did not consider variable reaction specificity for each starting material; thus, the amidation reactions may be biased toward more reactive head groups.

To add confidence in the library, we filtered the data through SIRIUS (16), a software which utilizes isotope patterns and fragmentation trees to aid in substructure annotation, to ensure the MS/MS data could be explained by the assigned structures. Manual inspection of diagnostic fragment peaks generated an MS/MS library of 436 unique compounds represented by 726 MS/MS spectra. The distribution of adducts was [M + H]^+^ (*n* = 416), [M + Na]^+^ (*n* = 147), [M + K]^+^ (*n* = 25), [M + NH_4_]^+^ (*n* = 53) adducts and MS/MS of water loss in-source fragment [M-H_2_O + H]^+^ (*n* = 122), which were added to the public MS/MS libraries in GNPS and in Zenodo (17) as the “3-HYDROXY-ACYL-AMIDES-LIBRARY.” We manually curated the MS/MS spectra by looking for the diagnostic peaks of the head group. For example, to validate the spectra for glutamic acid conjugated to a C12:0 chain (12 carbons and 0 double bonds), we used data from MS/MS spectra on the GNPS library where two public spectra for glutamic acid show consensus fragment peaks of 84.04, 102.06, and 130.05. Since these three peaks were also found in the spectra assigned to the 3-hydroxy-conjugate (3-hydroxylauroyl glutamic acid), they were retained during filtering. Most of these *N*-acyl amides for which we created a reference library did not only have MS/MS spectra in the public domain but were also largely absent from the most comprehensive structural databases, such as LipidMAPS (18) and PubChem (19).

Only three of these *N*-acyl amides were found in LipidMAPS (18), the leading structural resource in the lipid field. These were 3-hydroxyoctadecanoyl ornithine, 3-hydroxyhexadecanoyl ornithine, and 3-hydroxytetradecanoyl glycine. Only 79 were found in PubChem (19), and only one, 3-hydroxyoctadecanoyl glycine, has previously been reported to be detected by the Human Metabolome Database (HMDB) (20), and there was none in the MassBank of North America (MONA) (21) (Table S2). This new spectral resource represents a unique opportunity to reveal detected but not yet annotated biochemistry in the public domain data. We also aim to improve annotation in future LC-MS/MS-based untargeted metabolomics studies, leading to an enhanced understanding of biology.

**Fig 2:**
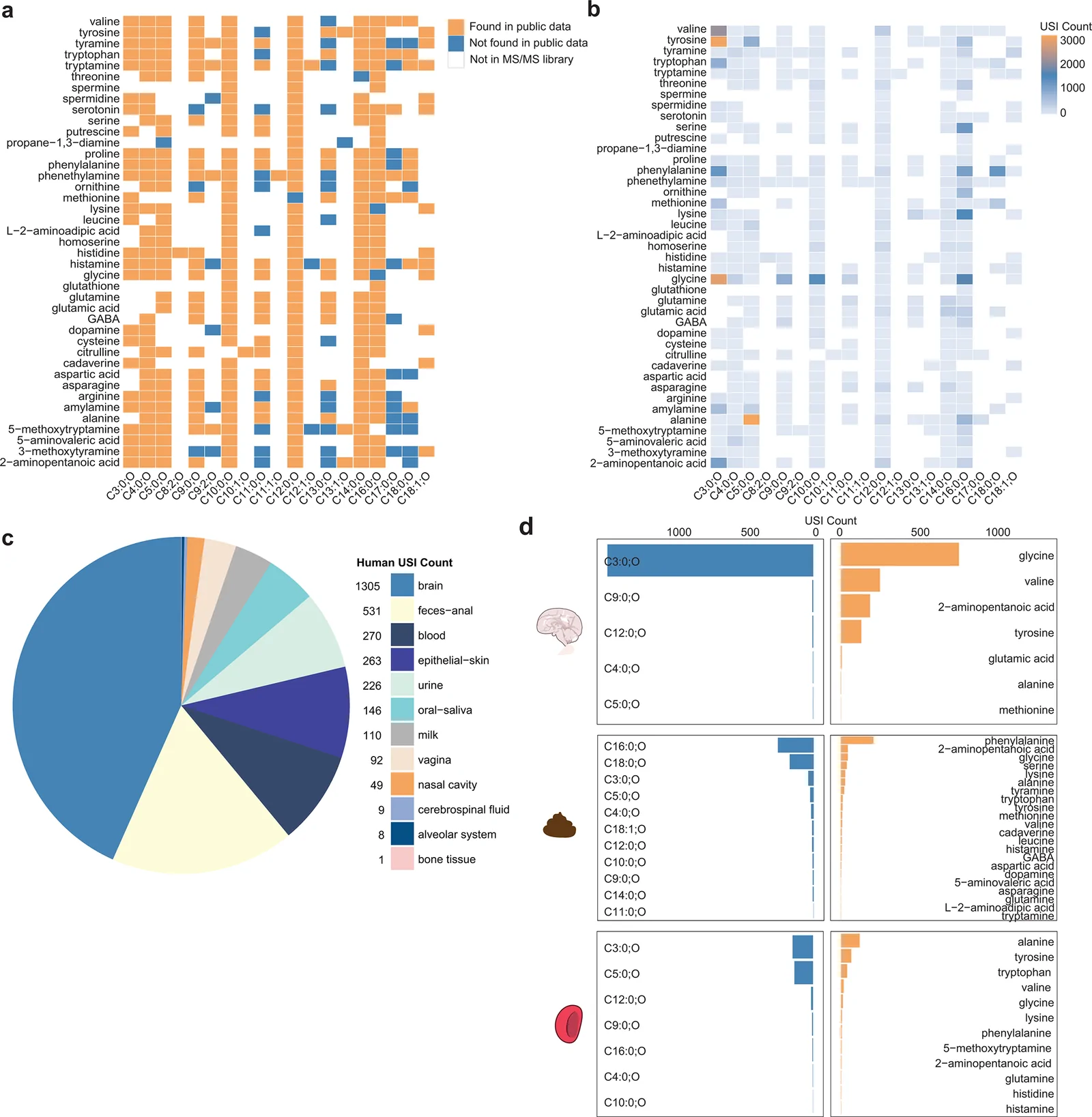
Pan-repository analysis of the 3-hydroxy *N*-acyl amide library. (**a**) Heatmap showing presence/absence of 3-hydroxy acyl amide combinations of differing tails (chain lengths) and heads (amines). Lipid chains are annotated as the number of carbons and number of unsaturations in the chain, followed by annotation of a hydroxyl modification. The *y*-axis shows head groups used in synthesis ordered alphabetically, and the *x-*axis shows chain lengths used in synthesis ordered by length. A colored square indicates that a library spectrum exists for a unique head–tail combination. A blue square indicates no spectral match (minimum cosine of 0.7) exists within public data repositories for that acyl amide combination. An orange square indicates at least one spectral match (minimum cosine of 0.7) within public data. A white square indicates that the head–tail combination is not present in the MS/MS library. (**b**) Heatmap represents the number of MASST spectral matches (minimum cosine of 0.7) to each library spectrum subset by head and tail. (**c**) Pie chart shows the distribution of the entire 3-hydroxy acyl amide library against all public data, with metadata labeled as “homo sapiens” with sample type (UBERONBodyPartName) information found in Pan-ReDU (4). The sample type with the most spectral matches is brain, followed by feces-anal (concatenation of samples labeled feces or anal tissue). (**d**) Bar charts show the number of spectral matches per tail and head substructure for the top three most annotated sample types—brain, feces-anal, and blood (includes serum and plasma).

To provide evidence of their presence in biology, the reference library consisting of 763 MS/MS spectra underwent reverse metabolomics analysis (Fig. 2a). Using reverse metabolomics, we found 161,626 spectral matches in the public domain with a minimum cosine similarity of 0.7 using a minimum of three matched ions, representing 334 unique 3-hydroxy *N*-acyl lipids (Fig. 2b). These were matched to data from 1,255 studies (GNPS/MassIVE *n* = 1,192, Metabolights *n* = 45, Metabolomics Workbench *n* = 19) in 54,744 data files. Of the unique data files, 1,025 match to Metabolights, 59 to Metabolomics Workbench, and 53,660 to GNPS/MassIVE (Table S3). Recently, we investigated what proportion of public metabolomics data among these three repositories could be annotated through MS/MS spectral matching by the GNPS public library (4). Of more than 1.7 billion MS/MS spectra, 142,538,419 had a spectral match (8.1%). We queried this recently developed SQLite database of these spectral matches to show the 3-hydroxy *N*-acyl amide library corresponded to 0.03% of these annotations. Similarly, common structural classes, including “phenylpropanoic acids” (as described by NPClassyfire), correspond to 0.02% of annotations, and “heteroaromatic compounds” correspond to 0.02%, whereas more highly annotated classes, such as “indoles and derivatives” or “seroid and steroid derivatives” are 1.19% or 3.55%, respectively (Table S4).

The proposed origin of these lipids is β-oxidation or classical acetate-mediated lipid assembly, where fatty acids are built up or broken down in two-carbon units (14, 22). When comparing the unsaturated lipids to their saturated counterparts for which we had spectra available (e.g., 3OH-C10:0 to 3OH-C10:1), there was a decrease in numbers (matching unsaturated *n* = 3,933 and saturated *n* = 89,094). However, even chains (*n* = 45,531) showed similar prevalence to odd chains (*n* = 43,563) by spectral matching. A significant portion of the odd chains found were C3:0 (*n* = 17,893, representing 41% of the matches). It is likely that these conjugates’ lipids originate elsewhere, or the matches come from an isomer, such as lactate.

Among the samples in Pan-ReDU (23), the infrastructure which harmonizes metadata from four main mass spectral repositories into controlled ontologies, we obtained spectral matches to 3,010 human data files, 2,386 to rodents, 4,278 to plants (24), 6,505 to microbes (25), and 615 to food (26) (Table S5). The remaining data files belonged to other organisms or did not yet have computer-readable metadata. When comparing the number of unique compound matches to each domain, microbes had the largest number of matches to MS/MS of the 3-hydroxy *N*-acyl amide library, indicating that many of these molecules were likely of microbial origin.

Of the available data sets in rodents, the whole family of compounds was found in the blood (1,137 MS/MS matches, representing nine compounds), feces (401, 19 compounds), caecum (316 MS/MS spectra, eight compounds), ileum (144 MS/MS spectra, 10 compounds), and jejunum (130 MS/MS spectra, four compounds), among other tissue types (Table S6). In humans, MS/MS detection was in the brain (1,305 MS/MS spectra, representing nine compounds), feces and anal (531 MS/MS spectra, 42 compounds), blood (270 MS/MS spectra, 16 compounds), skin (263 MS/MS spectra, 14 compounds), and urine (146 MS/MS spectra, 12 compounds) (Fig. 2c and d; Table S7). In humans, the fecal samples had the highest diversity of 3-hydroxy *N*-acyl amides containing combinations of 11 3-hydroxy fatty acids and 22 amine head groups, whereas the brain had the most MS/MS matches with a unique combination of only five tails and seven heads through the overall repository-scale searches. It is important to note that the repository-scale analysis is based on spectral matching alone, and, therefore, other potential isomers might exist. To reach level 1 confidence according to the metabolomics standards initiative (15), we ran additional follow-up validation experiments utilizing identical and direct retention time and drift time comparisons between biological extracts and synthetic standards, in addition to spectral matching in select data sets.

### Bacterial origin of 3-hydroxy *N*-acyl amides

**Fig 3:**
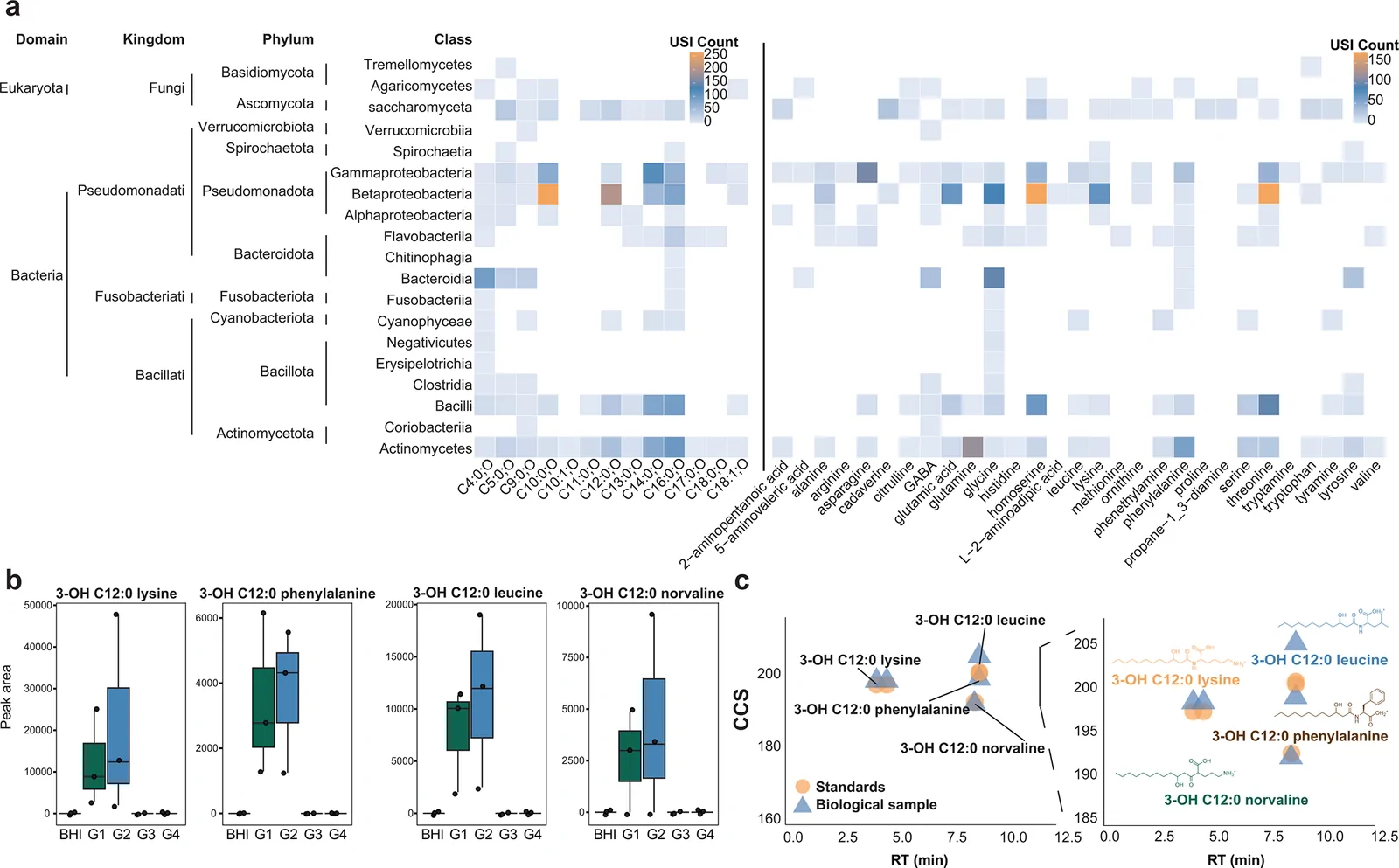
Bacterial origin of 3-hydroxy *N*-acyl amides. (**a**) Heatmap shows the number of USI (public MS/MS spectra), which had a microbeMASST (25) spectral match to the 3-hydroxy *N*-acyl amide library stratified by the head (amine) and tail (chain length) of the molecule. The *x*-axis is clustered based on chain length from smallest to largest (left panel) and amines by alphabetical (right panel). The *y*-axis is clustered by NCBI taxonomy and labeled from domain to class. (**b**) Boxplots show feature abundance of detected 3-hydroxy *N*-acyl amides within the synthetic microbial communities experiment at T = 72 h. BHI represents the media with the addition of 3-hydroxy lipids without any added bacteria. G1 represents fast-growing gut community members; G2 represents fast-medium growing; G3 represents medium-slow; and G4 represents slow growers. Fast and medium-fast growers show an increased abundance of the acyl amides compared to the media control. MS/MS mirror plots with the library can be found in Table S9. (**c**) Summary of retention time and CCS peaks of detected 3-hydroxy *N*-acyl amides within the microbial culturing experiment.

Searching our 3-hydroxy *N*-acyl amide library against public data revealed that most matches were against microbial samples, with many found in *Burkholderia*, *Bacteroides*, and other eubacteria (8, 9, 12) by using a domain-specific MASST, microbeMASST, with 61,000 taxonomically defined microbial data of monocultures (25). We found 6,505 MS/MS matches from the library to the microbial data, representing 135 3-hydroxy *N-*acyl amide spectra belonging to 111 unique compounds (Fig. 3a).

Many of the bacteria within microbeMASST, which had matches to the 3-hydroxy *N*-acyl amide library, belonged to classes of bacteria that are part of the human gut microbiome (e.g., Bacilli and Clostridia) (27, 28). We, therefore, hypothesized that many of the 3-hydroxy *N*-acyl amides detected in human or mouse (Tables S6 and S7) might be a product of microbial transformation of the proposed starting materials—3-hydroxy lipid chains, which are a product of endogenous lipid metabolism (Fig. 1a), and various head groups, including amino acids and other endogenous small amines. To test this hypothesis, we cultured defined gut communities (29) in brain heart infusion (BHI) media, which contains a broad range of substrates, including amino acids (30), and we added seven 3-hydroxy lipids, namely, C3:0, C4:0, C5:0, C11:0, C12:0, C13:0, and C16:0, based on their availability in our lab (Table S8). At 72 h, we observed that fast- (G1) and medium-fast (G2) grower communities were able to produce some 3-hydroxy *N*-acyl amides, most notably C12:0 chains, that were not observed in the control (medium alone) (Fig. 3b). We confirmed their identity through spectral matching, retention time, and drift time analysis (Fig. 3c), confirming the production of these molecules by bacteria.

### Potential origin of 3-hydroxy *N*-acyl amides in humans and their verification

**Fig 4:**
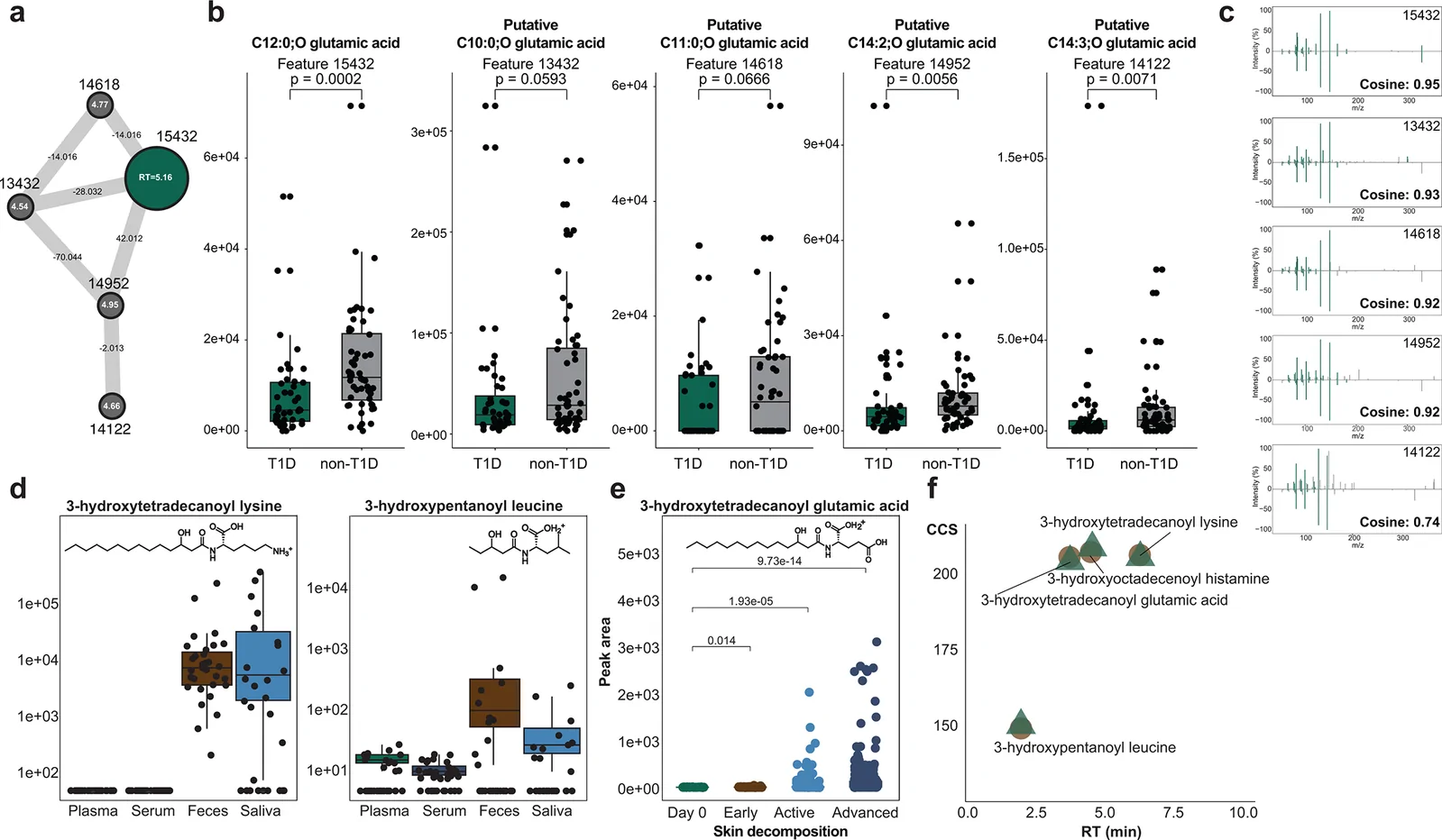
The production of 3-hydroxy *N*-acyl amides depends on energy metabolism, organ distribution, and microbial activity. (**a**) Network of features in the feature-based molecular networking shows related spectra of 3-hydroxylauroyl glutamic acid and their relative delta masses and retention times. (**b**) Univariate analysis of selected feature abundances from data set MSV000082261. Feature 15432 was annotated through MS/MS spectral matching (metabolomics initiate level 2) as 3-hydroxylauroyl glutamic acid, which is significantly (*P* < 0.05) depleted in urine samples labeled type one diabetes mellitus in the public metadata compared to the healthy samples. Features related to 3-hydroxylauroyl glutamic acid (13432, 14618, 14952, and 14122) are significantly (*P* < 0.05) depleted in the type 1 diabetes mellitus samples. Significance was assessed using the Wilcoxon rank-sum test. (**c**). MS/MS spectral mirror plots show high cosine similarity (cosine = 0.95) between feature 15432 and the synthetic spectra of 3-hydroxylauroyl glutamic acid (CCMSLIB00013641087). Other selected features in the molecular network (panel **b**) have high cosine similarity (>0.7) to feature 15432, showing their structural similarity. (**d**) Boxplots show feature abundance (log10 fold) of detected 3-hydroxy *N*-acyl amides, 3-hydroxytetradecanoyl lysine, and 3-hydroxypentanoyl leucine within different sample distributions in the MSV000094097 data set. (**e**) Boxplots show feature abundance of 3-hydroxytetradecanoyl glutamic acid within the MSV000084322 data set sampling skin swabs of outdoor body decomposition over time. 3-Hydroxytetradecanoyl glutamic acid was significantly increasing in skin decomposition (*P* < 0.05) with time. Significance was assessed using the Wilcoxon rank-sum test. The boxplots represent first (lower), interquartile range (IQR), and third (upper) quartile. (**f**) Summary of retention time and CCS peaks of detected 3-hydroxy *N*-acyl amides within all public data experiments. MS/MS mirror plots with the library can be found in Table S9.

We assessed the presence of these 3-hydroxy *N*-acyl amides within various human data sets found in Pan-ReDU in different biological conditions utilizing the MS/MS spectral library. We first investigated distribution in disease states, an approach to begin to understand the origin or function of these metabolites. There were 159 spectral matches to samples labeled as “inflammatory bowel disease” (IBD), 120 spectral matches to “primary bacterial infectious disease” (PBID), 60 to “diabetes mellitus” (diabetes), and others (Table S10). Investigation of the IBD and PBID data sets showed no metadata available to compare the abundance of the 3-hydroxy *N*-acyl amides between healthy and diseased participants. We thus targeted diabetes with data sets having metadata for urine samples from diabetics and non-diabetics. We performed feature extraction and feature-based molecular networking on the raw data available in MassIVE (MSV000082261). We found a spectral match to the library belonging to 3-hydroxylauroyl glutamic acid, then propagated annotations within the molecular network using spectral similarity, mass defects, and retention time, which putatively identified other hydroxy-lipid glutamic acid conjugates (Fig. 4a). Analysis of the peak areas of these features showed significant (*P* < 0.05) depletion of these metabolites in the urine of diabetic patients (Fig. 4b). Metabolic dysfunction, which may impact the synthesis or β-oxidation of fatty acids, is a characteristic of type 1 diabetes (31). If indeed the lipid substrates of 3-hydroxy *N-*acyl amides originate from this endogenous pathway, we would expect to see dysregulation of *N-*acyl amides in diabetics as we do here. Alternatively, some connections between microbiome composition and type 1 diabetes have been found, which may also contribute to this pattern (32–34).

Re-analysis of data samples from a cohort of osteoarthritis patients showed some tissue specificity of two newly annotated 3-hydroxy *N*-acyl amides (Fig. 4d). 3-Hydroxypentanoyl leucine was present in feces, serum, plasma, and saliva samples in this cohort. 3-Hydroxytetradecanoyl lysine, however, was present in many samples across only fecal and saliva samples. These molecules were also validated with retention time and drift time analysis (Fig. 4f). This tissue/biofluid specificity supports the conclusion that these molecules are indeed microbially made, as the gut and salivary microbiome are well documented (35). Reanalysis of a data set which sampled human cadavers’ skin showed the abundance of 3-hydroxytetradecanoyl glutamic acid significantly increased (*P* < 0.05) with advancement of skin decomposition (Fig. 4e). This metabolite was detected in the soil surrounding the cadavers with MS1, RT, and DT; however, its low peak intensity (<150) did not trigger MS/MS, limiting confidence in its identification and therefore the full interpretation of its presence in the soil. This molecule was validated with retention time and drift time analysis (Fig. 4f). 3-Hydroxytetradecanoyl glutamic acid increases with decomposition, mirroring the increase in microorganisms and their products (36), serving as further evidence for microbial origin of this molecule. Another undescribed molecule, 3-hydroxyoctadecenoyl histamine, was detected and validated in additional fecal samples as part of a cohort from the HIV Neurobehavioral Research Center (HNRC) (37), which aims to investigate neurological complications in people with HIV (Fig. S1b).

### Conclusion and future outlook

This study expanded the metabolomics annotation coverage for 3-hydroxy *N*-acyl amides, an underrepresented class of metabolites with known microbial origin and emerging biological importance in human disease. By targeting this class of compounds with known functional characteristics, such as amide linkages and β-hydroxylations, we were able to increase the known diversity of microbial-associated metabolites within humans. This generation of MS/MS spectra of previously unknown 3-hydroxy *N*-acyl amides followed by a readout across thousands of untargeted metabolomics studies and metadata enrichment of these studies generated functional and spatial hypotheses for these previously unknown compounds. We found that this class of 3-hydroxy *N*-acyl amides is found in hundreds of LC-MS/MS studies among diverse types of organisms and human samples, and we demonstrated that community cultures of gut bacteria, when given 3-hydroxy lipids as substrates, can lead to the production of *N*-acyl amides. Metabolic dysfunction influences the abundance of some 3-hydroxy *N*-acyl amides, and we provided insight into host-microbe metabolic interactions, though pathways are out of the scope of this study. Fundamentally, to propose such interactions and functions, we must be able to detect and annotate these compounds, which our public library will grant.

We shared 765 unique MS/MS spectra in this library, but thousands of other amine heads and unique lipid chain lengths remain uninvestigated. Further synthesis of such compounds or other *in silico* or computational techniques are needed to continue the investigation of such compounds. We hope that the workflow used here and the knowledge of the fragmentation patterns of these compounds can be leveraged to expand this understanding and increase general metabolomics annotations in a wide variety of experiments globally.

The library can be widely used and incorporated into untargeted metabolomics workflows and is made simple by its addition in the GNPS library and Zenodo. However, the discovery and characterization of these molecules through this resource have limitations.

Spectral matching by MS/MS is a putative identification of a metabolite, and orthogonal validation methods are necessary to confirm the identification of a metabolite within a sample, which we were able to do for nine new metabolites. However, we relied on spectral matching to publicly deposited data to provide evidence for the discovery of hundreds of other 3-hydroxy *N*-acyl amides, and false discovery rate estimation found that 7.3% of spectral matches made from this library could belong to a different ion in the GNPS library. This may be explained by the dominance of fragmentation ions that come from the amine head group (Fig. S1), which may be shared by other chemical structures.

A traditional target-decoy FDR could not be applied, as the approach poses an unsolved problem. Unlike peptides, small molecules cannot be shuffled or reversed to generate a valid decoy. Even in proteomics, decoy-based postprocessors have been shown to under-control the FDR (38). Our isomer-mapping estimate represents a conservative lower bound on this ambiguity, as it can only detect overlap with structures already present in the GNPS library.

Many ion forms, or in-source fragments such as water loss, of one molecule exist in mass spectrometry data. This library captured [M + H]^+^ for all library compounds and other common ions, such as [M + NH_4_]^+^, [M + Na]^+^, [M + H-H_2_O]^+^, or [M + K]^+^. This library cannot comprehensively annotate all ion forms of one molecule, some of which are biased to different analytical methods or instrumentation. Some computational methods have been developed to aid researchers with detection of all ion forms (39).

Similarly, with all the different instrumentation and analytical methods that exist for LC-MS/MS workflows (23), it is likely that the pan-repository search was not comprehensive of all 3-hydroxy *N*-acyl amides in the library, and the results are likely biased to similar methods used in the creation of the library. As such, results are likely to change with the increase of data deposition in the repositories used here. We recognize that obtaining spectra from multiple instruments or analytical methods, such as negative ionization mode, would improve the library and serve as a future direction. For example, based on available metadata shared on the instrumentation type used in the FASST search, 15.4% of MS/MS matches are to an Orbitrap instrument, whereas 1.7% to ToF, which may be explained by and highlight that the library which was created on an Orbitrap instrument can be more readily adopted by experimental methods also utilizing an Orbitrap. For the spectral matches we did obtain, interpretation of results is limited to the metadata which is publicly available or documented. We aim to use the metadata to give insight into the potential origin of the molecule by recognizing general patterns, such as several pieces of evidence of microbial production.

Lastly, we utilized a multiplex synthesis method for the high-throughput curation of the MS/MS spectral library. The extraction of the spectra and annotation of the 3-hydroxy *N*-acyl amides of the reaction mixtures was aided by two annotation tools and manual inspection of the spectra for the correct structural annotation. We did not produce isolated and purified compounds to validate the spectra, nor obtain NMR or other information to validate the synthesis.

## MATERIALS AND METHODS

### Multiplexed reactions

Separate four reagent substrate mixtures were created for the multiplex synthesis reactions (Table S11). In 20 mL of LC-MS grade water, we added 8 µmol of each amine and stored at –80°C until the reactions were performed. Either 1, 2, or 3 3-hydroxy fatty acids (1 eq.) and 2 mL of THF were added to a 20 mL scintillation vial with a magnetic stir bar. To this solution, neat ethyl chloroformate (1.2 eq.) and triethylamine (1.2 eq.) were subsequently added, and the reaction flask was placed in an ice water bath. In 1.5 h, 1 mL of amine mix was added, and the reaction continued to stir with ice cooling, allowing the ice to melt over 12 h. LC-MS/MS analyses were achieved using 1 µL of the reaction mixture diluted in 1 mL of LC-MS grade MeOH.

### Curation of the 3-hydroxy *N*-acyl amide library

The MS/MS library generation of the 3-hydroxy *N*-acyl amides was performed on a Thermo Scientific Orbitrap Exploris 240 Mass Spectrometer with a Thermo Vanquish UHPLC System. Samples were ionized by electrospray ionization (ESI) in positive mode using an H-ESI source (spray voltage: +3,500 V; sheath gas: 50; auxiliary gas: 10; sweep gas: 1; ion transfer tube temperature: 325°C; vaporizer temperature: 350°C). Chromatographic separation was carried out on a Phenomenex Polar C18 Column (2.6 µm particle size, 100 × 2.1 mm) using a 12-min linear gradient at 0.5 mL/min, with mobile phases consisting of H_2_O 0.1% formic acid (A) and ACN 0.1% formic acid (B). The gradient is as follows: 0–0.5 min, 5% B; 1.1 min, 25% B; 7.5 min, 40% B; 8.5 min, 99% B; 10.0 min, 99% B; 10.1 min, 5% B; and 12.0 min, 5% B. Full scans were acquired from *m*/*z* 80–1,000 at 120,000 resolution, with the automatic gain control (AGC) target set to standard. Data-dependent acquisition (DDA) selected top 10 precursors per cycle using an isolation window of *m*/*z* 1 and stepped HCD collision energies of 25, 35, and 50. MS/MS spectra were acquired at 60,000 resolving power with an AGC target set to standard and automatic maximum injection time. A 3 s exclusion duration and 3 ppm mass tolerance for dynamic exclusion were used. Isotope exclusion was enabled. Raw data were converted to open-source “.mzML” format in ProteoWizard (40) MSConvert program with centroid peak picking.

The spectral library was created in the reverse_metabolomics_create_library_workflow (7) in GNPS2, which searches for a given molecular formula in the mzML files. The molecular formulas were given as SMILES of the targeted compounds. Adducts of [M + H]^+^ were required for each compound, and additional adducts [M + Na]^+^, [M + NH_4_]^+^, [M-H_2_O + H]^+^, and [M + K]^+^ were included in the library if [M + H]^+^ or [M + NH_4_]^+^ was also included. Multiple adducts were included to enhance the rate of spectral matches to untargeted metabolomics data. The target list included 967 compounds, including the 798 acyl amides with some isomer forms when the amine head included more than one primary amine functional group. The formula search looked for ions within 10 ppm of the given formula mass, given that the intensity of the MS1 ion is a minimum of 5e4. Furthermore, the feature or MS/MS must have a minimum feature height of 1e5. This search found 536 unique target compounds, and 483 of the compounds had an [M + H]^+^ ion form. It further filters these matches with an “MS/MS explanation score,” which searches all fragments of the MS/MS for a formula-based substructure of the given parent formula with a minimum cosine level of 0.6. This filtering selected 443 unique compounds to create a library of 925 spectra.

Then, the spectra collected from the GNPS2 workflow are further validated for sub-structure annotation in SIRIUS 6.0.7 (16). Using the molecular formula prediction, CSI:finger ID, and database search of the spectral files using the same SMILES-based target list, SIRIUS was able to find 88.2% of the compounds from the GNPS2 workflow. It found 25 unique compounds that GNPS did not. Any compound found in both GNPS2 and SIRIUS was uploaded to the GNPS public library and Zenodo (17). Any feature found in only one of the programs was inspected manually. Acyl amides have a predictable fragmentation pattern in positive mode, which includes at least two high-intensity peaks from the head group. Using fragmentation patterns about the head group compounds in GNPS libraries, 32 of the 52 compounds not found by SIRIUS were recovered, and 11 of the 25 compounds not found by GNPS were recovered. A total of 313 compounds could not be detected in either GNPS2 or SIRIUS because of unsuccessful synthesis. This left 944 total spectra, which were then manually filtered for quality to create a spectral library of 764 spectra of 436 unique compounds with unique USI values. The filtered library spectra were uploaded to the public GNPS library through the batch annotator workflow on GNPS and is named 3-HYDROXY-ACYL-AMIDES-LIBRARY.

### Pan-repository compound search

We conducted a pan-repository-scale search using a fast MASST (41) (FASST) search, an updated and faster version of the Mass Spectrometry Search Tool (MASST), against all the public domain data that were indexed in GNPS/MassIVE, Metabolomics Workbench, and Metabolights. This search is based on the cosine similarity of the queried spectra against the ones from the public domain in GNPS/MassIVE, regardless of the instrument used for data acquisition. This search was conducted in the GNPS2 workflow fasst_batch_workflow which took the USI (42) of the library compounds as an input, requiring matches to public data to have a raw cosine similarity score >0.7, and precursor ion and fragment ion tolerances within 0.02 Da as done in previous pan-repository analyses. Upon the initial MASST query of the entire spectral library to all three public data repositories (metabolomics_pan_repo_index_nightly), there are 3,358,736 spectral matches. Then, inspection of the cosine similarity score of the raw matches kept 269,049 spectra with raw cosine >0.7 taken from the JSON of each spectral match on the metabolomics resolver (43) on GNPS.

Domain-specific MASSTs were conducted with the REST web API using metabolomics_pan_repo_index_nightly (23). One search curated three domain-specific output tables that contain spectral matches to specific data files in the public domain that can be mapped to the curated list of taxonomy/ontology-defined metadata: (1) microbeMASST (25), which merges the MASST spectral matches against a curated database of more than 60,000 LC-MS/MS files of microbial monocultures that were taxonomically defined; (ii) plantMASST (24),which merges the MASST matches against 19,075 LC-MS/MS files of plant extracts of taxonomically defined plants; and (iii) foodMASST (26), which merges MASST matches against ∼3,500 LC-MS/MS files of foods and beverages categorized within a food ontology collected as part of the Global FoodOmics project. We derived phylogeny from the National Center for Biotechnology Information (NCBI) taxonomy in microbiomeMASST by querying the NCBI API using R package Taxize (version 0.10.0). Creation of the tree was done through manual query of the NCBI taxonomy database.

To explore the molecules organ, tissue, and biofluid distribution in human and mice studies, we leveraged the Pan-ReDU curated metadata to filter for 9606|Homo sapiens or rodent-related data sets (“10088|Mus,” “10090|Mus musculus,” “10105|Mus minutoides,” “10114|Rattus,” “10116|Rattus norvegicus”) as written in the Reverse Metabolomics protocol (5). Packages used for data table manipulation included readr (version 2.1.5), stringr (version 1.5.2), dplyr (version 1.1.4), tibble (version 3.3.0), tidyr (version 1.3.1), and data.table (version 1.17.8). Heatmaps, bar plots, and pie charts of the pan-repository data were created in RStudio with the ggplot package (version 4.0.0).

### Reanalysis of public data

The 3OH-acyl amide library was used to analyze data sets MSV000082261 (diabetes), MSV000094097 (44) (osteoarthritis), MSV000092833 (37) (HNRC), and MSV000084322 (36) (dead bodies). For each data set, the files were downloaded from GNPS/MassIVE and processed in MZmine (39). The linked parameters and software version used for each study are available in Table S12. The corresponding “iimn” (ion identify molecular networking) files were used in the feature-based molecular networking available on GNPS2 with library search against 3-HYDROXY-ACYL-AMIDES-LIBRARY. The networking jobs are available in Table S13. The parameters for the match were set as 0.02 Da, and cosine similarity score was >0.7. The number of matching peaks was set to 2, as some of the spectra, like 3-hydroxybutyroyl glycine, or [M + Na]^+^ adducted ions have very few fragments in the MS/MS spectra. Manual inspection of the matched spectra was used to verify the library matches to the biological spectra, and further validation through retention time and drift time matching was used if we had access to the samples. Molecular networking on the GNPS2 interface was used to find chain length analogs to the compounds detected with the library search, as used in the analysis of MSV000082261 and were labeled as putative because they are an MSI level 2. The resulting feature tables were used for relative peak area analysis. Packages used for data table manipulation included readr (version 2.1.5), stringr (version 1.5.2), dplyr (version 1.1.4), tibble (version 3.3.0), tidyr (version 1.3.1), and data.table (version 1.17.8). Box plots and heatmaps were created using the ggplot package (version 4.0.0), ggsignif (version 0.6.4), and scales (version 1.4.0) in RStudio. Statistical significance was assessed using the Wilcoxon rank-sum test. All boxplots represent first (lower), interquartile range (IQR), and third (upper) quartile.

### Bacterial culture screening

A total of 80 strains of bacteria (Table S14) were inoculated from glycerol stocks and cultured anaerobically in filter-sterilized modified brain-heart infusion (BHI) broth (75% N_2_, 20% CO_2_, and 5% H_2_) (Table S15). Each strain was normalized to OD_600_ = 0.02 and combined into a Master Mix according to growth rate classifications: Group 1 (fast growers), Group 2 (medium-fast growers), Group 3 (medium-slow growers), and Group 4 (slow growers). The four groups were pooled together and diluted 1:10 to construct the full hcom (human gut synthetic community). The 3-hydroxy lipids were added at 100 µM to a shallow 96 well-plate followed by the microbial communities to be incubated anaerobically at 37°C for 72 h. After incubation, bacterial cells were extracted with a ratio sample to solvent of 1:4 with either 80% of pre-chilled MeOH/H_2_O with 1 µM sulfamethizole and incubated overnight at 4°C. Samples were centrifuged at 1,800 rpm for 10 min, and the supernatants were transferred to a new plate before being dry in a CentriVap overnight. Samples were stored at −80°C until LC-MS/MS analysis. Samples were resuspended in 50:50 MeOH/water with 1 µM sulfadimethoxine internal standard and run on an Orbitrap Exploris 240 instrument using electrospray ionization (ESI) with chromatographic separation through the Thermo Fisher Vanquish attached to a Phenomenex 2.6 µm Polar C18 100 × 2.1 mm column. The synthetic samples were eluted on a 12 min gradient with flow rate of 0.5 µ/min, and the mobile phase included H_2_O + 0.1% formic acid and ACN + 0.1% formic acid. Raw data were converted to open-source mzML format in ProteoWizard (40) MSConvert program. The resulting .mzML files were processed in MZmine4 (39) (hcomm_batch.mzbatch at https://github.com/victoriadeleray/3OH_N-acyl_amides). The resulting feature table and spectral list (available on https://github.com/victoriadeleray/3OH_N-acyl_amides as hcomm__iimn_gnps.csv and hcomm__iimn.mgf) were used for relative peak area analysis and annotation within the feature-based molecular networking workflow on GNPS2 (task id = abcfa3d422534d86b2e71a24efbd7fc6). The peak area table was filtered to remove blanks, and internal standards were used for quality control. Box plots and heatmaps were created using the ggplot package (version 4.0.0) and tidyverse package (version 2.0.0) in RStudio. The boxplots represent first (lower), interquartile range (IQR), and third (upper) quartile.

### Retention time and drift time analysis

The putative identifications (MS/MS spectral matching) of 3-hydroxy *N*-acyl amides in biological samples, of which we had available in our laboratory, were further validated in a secondary LC-IMS-MS/MS experiment where the fecal, microbial, and skin samples were analyzed in the same experimental conditions as the synthetic standards of the 3-hydroxy *N*-acyl amide annotations. The retention time (RT) analysis was run on the timsTOF Pro2 mass spectrometer (Bruker Daltonics) with chromatographic separation through the Agilent Infinity three ultra-high resolution liquid chromatography attached to a Phenomenex 2.6 µm Polar C18 100 × 2.1 mm column. Samples were eluted at 0.5 mL/min using the following gradient: 0–0.5 min, 5% B; 1.1 min, 25% B; 7.5 min, 40% B; 8.5 min, 99% B; 10.0 min, 99% B; 10.1 min, 5% B; and 12.0 min, 5% B. The instrument was operated in positive ESI with a capillary voltage of 4,500 V, end-plate offset of 500 V, nebulizer pressure of 2.2 bar, dry gas of 10.0 L/min, and dry gas temperature of 220°C. MS data were acquired from *m*/*z* 20–1,200 in PASEF mode. TIMS mobility was collected with a 1/*K*_0_ range of 0.10–1.50 V·s/cm², ramp time of 100 ms, accumulation time of 100 ms, and a duty cycle of 100%. External mass and mobility calibration were performed using the Bruker ESI Tuning Mix (ESI-TOF CCS compendium). Extracted ion mobilograms of the [M + H]^+^ of each targeted ion were exported through the Bruker DataAnalysis 6.1 software. Raw data were converted to open-source “.mzML” format in ProteoWizard (40) MSConvert program (64-bit encoding, vendor peakPicking filter for MS1–MS2, zlib compression enabled) for input in the GNPS dashboard to export extracted ion chromatograms for RT plots. A confident CCS match was reported if the CCS of the metabolite in the sample was within 2% of the standard, and a confident retention time match was reported if the elution time of the metabolite in the sample was within 0.1 minutes of the standard.

### Spectral ambiguity estimation

To estimate which FASST matches can be annotated with more than one known structure, we ran the library search workflow on GNPS2 to calculate the cosine similarity between the 3-hydroxy *N*-acyl amide library MS/MS against the existing GNPS library MS/MS to determine which 3-hydroxy *N*-acyl amide spectra had a minimum cosine of 0.7 and three minimum matched peaks to a spectra derived from a different ion. There were a total of 62 spectral matches after filtering out “candidate,” “putative,” or “possible” structures that exist in the GNPS library. Of these, seven matches were to spectra labeled with the same parent structure, and 24 matches were spectral matches between the same ions, for example, 3-hydroxytetradecanoyl glycine [M + H-H_2_O]^+^ matches Gly-C14:1 [M + H]^+^. Additionally, eight matches were to structural isomers, such as a spectral match between 3-hydroxylauroyl threonine and 3-hydroxydodecanoyl homoserine, or 10-hydroxydecanoyl-3-methoxytyramine matched 3-hydroxydecanoyl-3-methoxytyramine. At the MS/MS level, with our chosen analytical methods, we cannot differentiate isomers threonine and homoserine or 3-hydroxydecanoic acid and 10-hydroxydecanoic acid. The remaining 23 matches were to spectra from non-isomeric structures, such as the match between 3-hydroxytetradecanoyl tyramine [M + H]^+^ and Ser-C16:1 [M + H]^+^ (Table S16).

These results were then mapped to the FASST results (pan repository spectral matches) to estimate the percent of spectral matches with known structural ambiguity, yielding that 5.7% of such results come from spectra that potentially map non-isomeric structures, and 7.3% potentially map isomeric and non-isomeric structures.

An important constraint is that a formal decoy-based FDR could not be pursued, as constructing decoy spectra or decoy structures for small molecules remains an unsolved problem distinct from peptide-based proteomics, where a decoy can be generated directly from the target sequence by shuffling or reversal. Even within proteomics, where this generative null is well defined, decoy-based postprocessors, such as Percolator and PeptideProphet, have been shown to systematically exceed their nominal FDR threshold, particularly as the in-sample search space narrows, indicating that target-decoy control can be imperfect, even under near-ideal conditions (38). Metabolomics-specific decoy-spectrum methods, such as passatutto, avoid generating decoy structures for the same reason, but their own validation showed that FDR estimates become unreliable precisely when a structural isomer with near-identical fragmentation exists in the reference library (45), the same phenomenon underlying the isomer-driven ambiguity reported here. Even if we wanted to use spectrum decoy it does not scale at the repository level. We therefore consider our isomer-mapping approach, while not a formal FDR, consistent with the best available evidence on where decoy-based estimation in this field breaks down.

## Supplementary Material

Reviewer comments

## Data Availability

Code, data tables, and other supplemental material can be found at https://github.com/victoriadeleray/3OH_N-acyl_amides. The public spectral library can be found and downloaded at https://external.gnps2.org/gnpslibrary as 3-HYDROXY-ACYL-AMIDES-LIBRARY or Zenodo (17) at https://zenodo.org/records/17488401. Raw data from the multiplexed synthesis reactions and retention time analyses can be accessed in GNPS/MassIVE at https://massive.ucsd.edu/ProteoSAFe/dataset.jsp?task=a682622f3b464c7da6b130c62cd4c32e under accession MSV000095216.

## SUPPLEMENTAL MATERIAL

Supplemental material msystems.00839-26-s0001.pdf Supplemental tables and figure.

OPEN PEER REVIEW reviewer-comments.pdf An accounting of the reviewer comments and feedback.

ASM does not own the copyrights to Supplemental Material that may be linked to, or accessed through, an article. The authors have granted ASM a non-exclusive, world-wide license to publish the Supplemental Material files. Please contact the corresponding author directly for reuse.
