# Supplementary material for: Synthesis-driven reverse metabolomics reveals 3-hydroxy N-acyl amides as gut microbial molecules
Source: mSystems. 2026 Sep 24;11(10):e00839-26. doi: 10.1128/msystems.00839-26 (PMC13712783; doi:10.1128/msystems.00839-26)
Supplement: Supplementary file 1 [file msystems.00839-26-s0001.pdf]

**Supplementary Table Legend**	
Table S1	Multiplex synthesis reagents and reaction mixtures
Table S2	3-hydroxy N-acyl amide structures in public databases
Table S3	Number of public datasets with 3-hydroxy N-acyl amides
Table S4	FASST Records SQL database by NPClassifier
Table S5	Number of public files with organism-level metadata containing 3-hydroxy N-acyl amides
Table S6	Organ distribution in rodents by indexed spectral matches
Table S7	Organ distribution in humans by indexed spectral matches
Table S8	3-hydroxy N-acyl amide substrates used in culturing
Table S9	MS2 mirror plots containing 3-hydroxy N-acyl amide library matches
Table S10	Disease distribution in humans by indexed spectral matches
Table S11	Multiplex synthesis reagents by mixture
Table S12	MZmine information
Table S13	GNPS2 Molecular networking of publically downloaded data
Table S14	List of all strains used in community culturing experiment
Table S15	Culturing media recipe
Table S16	GNPS2 library search results for spectral ambiguity estimation

Table S1

Multiplex synthesis reagents and reaction mixtures

Reaxys codes to cell	C3:0	C4:0	C5:0	C9:0	C10:0	C11:0	C12:0	C13:0	C14:0	C16:0	C17:0	C18:0	C10:1	C11:1	C12:1	C13:1	C14:1	C18:1	C8:2	C9:2
Leucine	VD-49	KV-13	KV-13	KV-20	VD-53	KV-20	VD-55	KV-20	VD-61	VD-65	KV-20	KV-20	neuro	neuro	neuro	VD-72	neuro	VD-71	neuro	neuro
histamine	VD-49	KV-13	KV-13	KV-20	VD-53	KV-20	VD-55	KV-20	VD-61	VD-65	KV-20	KV-20	neuro	neuro	neuro	VD-72	neuro	VD-71	neuro	neuro
5-methoxytryptamine	VD-49	KV-13	KV-13	KV-20	VD-53	KV-20	VD-55	KV-20	VD-61	VD-65	KV-20	KV-20	neuro	neuro	neuro	VD-72	neuro	VD-71	neuro	neuro
tryptamine	VD-49	KV-13	KV-13	KV-20	VD-53	KV-20	VD-55	KV-20	VD-61	VD-65	KV-20	KV-20	neuro	neuro	neuro	VD-72	neuro	VD-71	neuro	neuro
tyramine	VD-49	KV-13	KV-13	KV-20	VD-53	KV-20	VD-55	KV-20	VD-61	VD-65	KV-20	KV-20	neuro	neuro	neuro	VD-72	neuro	VD-71	neuro	neuro
serotonin	VD-49	KV-13	KV-13	KV-20	VD-53	KV-20	VD-55	KV-20	VD-61	VD-65	KV-20	KV-20	neuro	neuro	neuro	VD-72	neuro	VD-71	neuro	neuro
5-aminovaleric acid	VD-49	KV-13	KV-13	KV-20	VD-53	KV-20	VD-55	KV-20	VD-61	VD-65	KV-20	KV-20	neuro	neuro	neuro	VD-72	neuro	VD-71	neuro	neuro
gaba	VD-49	KV-13	KV-13	KV-20	VD-53	KV-20	VD-55	KV-20	VD-61	VD-65	KV-20	KV-20	neuro	neuro	neuro	VD-72	neuro	VD-71	neuro	neuro
3-methoxy tyramine	VD-49	KV-13	KV-13	KV-20	VD-53	KV-20	VD-55	KV-20	VD-61	VD-65	KV-20	KV-20	neuro	neuro	neuro	VD-72	neuro	VD-71	neuro	neuro
dopamine	VD-49	KV-13	KV-13	KV-20	VD-53	KV-20	VD-55	KV-20	VD-61	VD-65	KV-20	KV-20	neuro	neuro	neuro	VD-72	neuro	VD-71	neuro	neuro
citrulline	VD-48	KV-14	KV-14	KV-20	VD-52	KV-20	VD-57	KV-20	VD-62	VD-66	KV-20	KV-20	misc	misc	misc	VD-72	misc	VD-71	misc	misc
L-2-aminoadipic acid	VD-48	KV-14	KV-14	KV-20	VD-52	KV-20	VD-57	KV-20	VD-62	VD-66	KV-20	KV-20	misc	misc	misc	VD-72	misc	VD-71	misc	misc
Glutathione	VD-48	KV-14	KV-14	KV-20	VD-52	KV-20	VD-57	KV-20	VD-62	VD-66	KV-20	KV-20	misc	misc	misc	VD-72	misc	VD-71	misc	misc
ornithine	VD-48	KV-14	KV-14	KV-20	VD-52	KV-20	VD-57	KV-20	VD-62	VD-66	KV-20	KV-20	misc	misc	misc	VD-72	misc	VD-71	misc	misc
homoserine	VD-48	KV-14	KV-14	KV-20	VD-52	KV-20	VD-57	KV-20	VD-62	VD-66	KV-20	KV-20	misc	misc	misc	VD-72	misc	VD-71	misc	misc
(S)-2-aminopentanoic	VD-48	KV-14	KV-14	KV-20	VD-52	KV-20	VD-57	KV-20	VD-62	VD-66	KV-20	KV-20	misc	misc	misc	VD-72	misc	VD-71	misc	misc
propane-1,3-diamine	VD-48	KV-14	KV-14	KV-20	VD-52	KV-20	VD-57	KV-20	VD-62	VD-66	KV-20	KV-20	misc	misc	misc	13_1_misc	misc	VD-71	misc	misc
Putrescine	VD-48	KV-14	KV-14	KV-20	VD-52	KV-20	VD-57	KV-20	VD-62	VD-66	KV-20	KV-20	misc	misc	misc	VD-72	misc	VD-71	misc	misc
spermine	VD-48	KV-14	KV-14	KV-20	VD-52	KV-20	VD-57	KV-20	VD-62	VD-66	KV-20	KV-20	misc	misc	misc	VD-72	misc	VD-71	misc	misc
Amylamine	VD-50	KV-15	KV-15	KV-20	VD-54	KV-20	VD-56	KV-20	VD-60	VD-64	KV-20	KV-20	liq	liq	liq	VD-72	liq	VD-71	liq	liq
cadaverine	VD-50	KV-15	KV-15	KV-20	VD-54	KV-20	VD_56	KV-20	VD-60	VD-64	KV-20	KV-20	liq	liq	liq	VD-72	liq	VD-71	liq	liq
Spermidine	VD-50	KV-15	KV-15	KV-20	VD-54	KV-20	VD-56	KV-20	VD-60	VD-64	KV-20	KV-20	liq	liq	liq	VD-72	liq	VD-71	liq	liq
Phenethylamine	VD-50	KV-15	KV-15	KV-20	VD-54	KV-20	VD-56	KV-20	VD-60	VD-64	KV-20	KV-20	liq	liq	liq	VD-72	liq	VD-71	liq	liq
Valine	VD-47	KV-12	KV-12	KV-19	VD-51	KV-19	VD-58	KV-19	VD-59	VD-63	KV-19	KV-19	p	p	p	VD-72	p	VD-71	p	p
Tyrosine	VD-47	KV-12	KV-12	KV-19	VD-51	KV-19	VD-58	KV-19	VD-59	VD-63	KV-19	KV-19	p	p	p	VD-72	p	VD-71	p	p
Tryptophan	VD-47	KV-12	KV-12	KV-19	VD-51	KV-19	VD-58	KV-19	VD-59	VD-63	KV-19	KV-19	p	p	p	VD-72	p	VD-71	p	p
Threonine	VD-47	KV-14	KV-12	KV-19	VD-51	KV-19	VD-58	KV-19	VD-59	VD-63	KV-19	KV-19	p	p	p	VD-72	p	VD-71	p	p
Serine	VD-47	KV-12	KV-12	KV-19	VD-51	KV-19	VD-58	KV-19	VD-59	VD-63	KV-19	KV-19	p	p	p	VD-72	p	VD-71	p	p
Proline	VD-47	KV-12	KV-12	KV-19	VD-51	KV-19	VD-58	KV-19	VD-59	VD-63	KV-19	KV-19	p	p	p	VD-72	p	VD-71	p	p
Phenylalanine	VD-47	KV-12	KV-12	KV-19	VD-51	KV-19	VD-58	KV-19	VD-59	VD-63	KV-19	KV-19	p	p	p	VD-72	p	VD-71	p	p
Methionine	VD-47	KV-12	KV-12	KV-19	VD-51	KV-19	VD-58	KV-19	VD-59	VD-63	KV-19	KV-19	p	p	p	VD-72	p	VD-71	p	p
Lysine	VD-47	KV-12	KV-12	KV-19	VD-51	KV-19	VD-58	KV-19	VD-59	VD-63	KV-19	KV-19	p	p	p	VD-72	p	VD-71	p	p
Histidine	VD-47	KV-12	KV-12	KV-19	VD-51	KV-19	VD-58	KV-19	VD-59	VD-63	KV-19	KV-19	p	p	p	VD-72	p	VD-71	p	p
Glycine	VD-47	KV-12	KV-12	KV-19	VD-51	KV-19	VD-58	KV-19	VD-59	VD-63	KV-19	KV-19	p	p	p	VD-72	p	VD-71	p	p
Glutamic Acid	VD-47	KV-12	KV-12	KV-19	VD-51	KV-19	VD-58	KV-19	VD-59	VD-63	KV-19	KV-19	p	p	p	VD-72	p	VD-71	p	p
Glutamine	VD-47	KV-12	KV-12	KV-19	VD-51	KV-19	VD-58	KV-19	VD-59	VD-63	KV-19	KV-19	p	p	p	VD-72	p	VD-71	p	p
Cysteine	VD-47	KV-12	KV-12	KV-19	VD-51	KV-19	VD-58	KV-19	VD-59	VD-63	KV-19	KV-19	p	p	p	VD-72	p	VD-71	p	p
Aspartic Acid	VD-47	KV-12	KV-12	KV-19	VD-51	KV-19	VD-58	KV-19	VD-59	VD-63	KV-19	KV-19	p	p	p	VD-72	p	VD-71	p	p
Asparagine	VD-47	KV-12	KV-12	KV-19	VD-51	KV-19	VD-58	KV-19	VD-59	VD-63	KV-19	KV-19	p	p	p	VD-72	p	VD-71	p	p
Arginine	VD-47	KV-12	KV-12	KV-19	VD-51	KV-19	VD-58	KV-19	VD-59	VD-63	KV-19	KV-19	p	p	p	VD-72	p	VD-71	p	p
Alanine	VD-47	KV-12	KV-12	KV-19	VD-51	KV-19	VD-58	KV-19	VD-59	VD-63	KV-19	KV-19	p	p	p	VD-72	p	VD-71	p	p
isoelucine		KV-12	KV-12																	

**Table S2**					
**3-hydroxy N-acyl amide structures in public databases**					
database	id	name			
lipidmaps	LMFA08020331	3-hydroxyhexadecanoyl glycine			
lipidmaps	LMFA08020332	3-hydroxytetradecanoyl glycine			
lipidmaps	LMFA08020333	3-hydroxyoctadecenoyl glycine			
lipidmaps	LMFA08020334	3-hydroxyhexadecenoyl glycine			
lipidmaps	LMFA08020225	3-hydroxyoctadecenoyl ornithine			
lipidmaps	LMFA08020242	3-hydroxyhexadecenoyl ornithine			
pubchem	101332887	3-hydroxytetradecanoyl leucine			
pubchem	101332888	3-hydroxytetradecanoyl aspartic acid			
pubchem	101352190	3-hydroxyoctadecanoyl ornithine			
pubchem	10512346	3-hydroxypropionyl leucine			
pubchem	10545348	3-hydroxyheptadecanoyl glycine			
pubchem	10711444	3-hydroxyhexadecanoyl glycine			
pubchem	107802	3-hydroxypentanoic acid			
pubchem	117874440	3-hydroxybutyroyl lysine			
pubchem	126726083	3-hydroxypropionyl alanine			
pubchem	130265970	3-hydroxypropionyl tyramine			
pubchem	131446863	3-hydroxypropionyl proline			
pubchem	137374025	3-hydroxyhexadecanoyl lysine			
pubchem	137628362	3-hydroxytetradecanoyl phenylalanine			
pubchem	137628488	3-hydroxyoctadecanoyl glycine			
pubchem	139391005	3-hydroxybutyroyl proline			
pubchem	139391011	3-hydroxybutyroyl serine			
pubchem	139391012	3-hydroxybutyroyl leucine			
pubchem	139391028	3-hydroxybutyroyl aspartic acid			
pubchem	139391050	3-hydroxybutyroyl histidine			
pubchem	139391131	3-hydroxybutyroyl phenylalanine			
pubchem	139404583	3-hydroxybutyroyl cysteine			
pubchem	139404695	3-hydroxybutyroyl asparagine			
pubchem	139404700	3-hydroxybutyroyl valine			
pubchem	140241936	3-hydroxyhexadecanoyl homoserine			
pubchem	14308140	3-hydroxypropionyl histidine			
pubchem	146240631	3-hydroxydecanoyl glycine			
pubchem	146240737	3-hydroxyundecanoyl glycine			
pubchem	146240792	3-hydroxydecanoyl leucine			
pubchem	146241018	3-hydroxynonanoyl glycine			
pubchem	146824820	3-hydroxypropionyl putrescine			
pubchem	152439931	3-hydroxybutyroyl threonine			
pubchem	15561476	3-hydroxypropionyl phenethylamine			
pubchem	156261173	3-hydroxypropionyl phenylalanine			
pubchem	157667062	3-hydroxybutyroyl tryptophan			

pubchem	172437676	3-hydroxybutyroyl 5-methoxytryptamine		
pubchem	21126063	3-hydroxydecanoyl serine		
pubchem	21337744	3-hydroxybutyroyl homoserine		
pubchem	441	3-hydroxybutyric acid		
pubchem	5282907	3-hydroxyoctadecanoic acid		
pubchem	53643105	3-hydroxypentanoyl leucine		
pubchem	53703432	3-hydroxybutyroyl alanine		
pubchem	53847138	3-hydroxylauroyl serine		
pubchem	54372927	3-hydroxyhexadecanoyl ornithine		
pubchem	54455579	3-hydroxypentanoyl homoserine		
pubchem	55286843	3-hydroxypropionyl glycine		
pubchem	57359503	3-hydroxy-4-decenoic acid		
pubchem	57359506	3-hydroxy-6-dodecenoic acid		
pubchem	57374116	3-hydroxy-4,6-nonadienoic acid		
pubchem	57374125	3-hydroxy-8-undecenoic acid		
pubchem	6710022	3-hydroxybutyroyl tyrosine		
pubchem	6710024	3-hydroxydecanoyl tyrosine		
pubchem	6710026	3-hydroxylauroyl tyrosine		
pubchem	6710028	3-hydroxytetradecanoyl tyrosine		
pubchem	6710030	3-hydroxyhexadecanoyl tyrosine		
pubchem	69602938	3-hydroxybutyroyl phenethylamine		
pubchem	73240735	3-hydroxybutyroyl tyramine		
pubchem	75069346	3-hydroxypentanoyl tyramine		
pubchem	85197100	3-hydroxytetradecanoyl glycine		
pubchem	87567267	3-hydroxypropionyl valine		
pubchem	87908763	3-hydroxybutyroyl amylamine		
pubchem	88613386	3-hydroxydecanoyl glutamine		
pubchem	89458166	3-hydroxybutyroyl glycine		
pubchem	90240727	3-hydroxyundecanoyl glutamine		
pubchem	90241098	3-hydroxylauroyl glutamine		
pubchem	90241245	3-hydroxynonanoyl glutamine		
pubchem	90241414	3-hydroxybutyroyl glutamine		
pubchem	90258176	3-hydroxyundecanoyl glutamic acid		
pubchem	90258193	3-hydroxybutyroyl glutamic acid		
pubchem	90258297	3-hydroxynonanoyl glutamic acid		
pubchem	90258356	3-hydroxylauroyl glutamic acid		
pubchem	90258443	3-hydroxydecanoyl glutamic acid		
pubchem	90258666	3-hydroxypentanoyl glutamic acid		
pubchem	90258771	3-hydroxypentanoyl glutamine		
pubchem	90425375	3-hydroxytetradecanoyl glutamic acid		
pubchem	90745071	3-hydroxylauroyl homoserine		
pubchem	91030869	3-hydroxytetradecanoyl homoserine		
pubchem	91071146	3-hydroxydecanoyl homoserine		

pubchem	94252202	3-hydroxypropionyl amylamine		
pubchem	94261118	3-hydroxypropionyl tryptamine		
GNPS	CCMSLIB000100130	(3-hydroxydodecanoyl)homoserine		
GNPS	CCMSLIB000100130	(3-hydroxytetradecanoyl)homoserine		
GNPS	CCMSLIB000100130	(3-hydroxytetradecanoyl)homoserine		
GNPS	CCMSLIB000101294	(3-hydroxydecanoyl)homoserine		
GNPS	CCMSLIB000046842	N-(3-hydroxytetradecanoyl)-L-phenylalanine		
GNPS	CCMSLIB000046792	Commendamide		
GNPS	CCMSLIB000162115	cadaverine_3-hydroxy_tetradecanoic_acid [CCS=203.99557495117188]		

Table S3		
**Number of public datasets with 3-hydroxy N-acyl amides**		
**n_distinct(Dataset)**	**Database**	
1192	MassIVE	
45	Metabolights	
19	Workbench	

Table S4				
**FASST Records SQL database by NPClassifier**				
level	annotation	n_matches	percent_of_all_annotated_masst	
class	NO_CLASS	711251626	59.06	
class	Fatty Acyls	108183195	8.98	
class	Carboxylic acids and	82842235	6.88	
class	Organooxygen comp	50803288	4.22	
class	Benzene and substitu	43823654	3.64	
class	Steroids and steroid d	42697728	3.55	
class	Flavonoids	41182984	3.42	
class	Indoles and derivative	14289798	1.19	
class	Organonitrogen comp	13183818	1.09	
class	Prenol lipids	9803150	0.81	
class	Organic phosphoric a	7527299	0.63	
class	Cinnamic acids and d	6788188	0.56	
class	Glycerophospholipids	6537006	0.54	
class	Imidazopyrimidines	5619818	0.47	
class	Phenols	5473772	0.45	
class	Dibenzocycloheptene	3496955	0.29	
class	Purine nucleosides	3328995	0.28	
class	Glycerolipids	2753753	0.23	
class	Pyridines and derivat	2322068	0.19	
class	Isoflavonoids	2263516	0.19	
class	Hydroxy acids and de	2066848	0.17	
class	Azoles	2038572	0.17	
class	Quinolines and deriva	1871832	0.16	
class	Pteridines and deriva	1775909	0.15	
class	5-deoxyribonucleosid	1560231	0.13	
class	Cinchona alkaloids	1358164	0.11	
class	Piperidines	1344037	0.11	
class	Purine nucleotides	1317402	0.11	
class	Thienopyridines	1290988	0.11	
class	Coumarins and deriva	1239314	0.1	
class	Lactones	1111162	0.09	
class	Sulfinic acids and der	1073051	0.09	
class	Organic sulfuric acids	964874	0.08	
class	Oxepanes	942538	0.08	
class	Organometalloid com	890844	0.07	
class	Macrolactams	888903	0.07	
class	Naphthalenes	799955	0.07	
class	Triazines	757355	0.06	
class	Benzofurans	741415	0.06	
class	Phenol ethers	738247	0.06	

class	Carboximidic acids a	668285	0.06	
class	Pyrimidine nucleotide	660927	0.05	
class	Diazines	660677	0.05	
class	Sphingolipids	647598	0.05	
class	Peptidomimetics	646764	0.05	
class	Diarylheptanoids	632015	0.05	
class	Harmala alkaloids	535595	0.04	
class	Oxazinanes	518858	0.04	
class	Pyrimidine nucleosid	461667	0.04	
class	Benzopyrans	458204	0.04	
class	Linear 1,3-diarylprop	418703	0.03	
class	Macrolides and analo	360524	0.03	
class	Pyrans	360143	0.03	
class	Kavalactones	331540	0.03	
class	Pyrazolopyrimidines	325441	0.03	
class	Stilbenes	303262	0.03	
class	Lignan glycosides	292148	0.02	
class	Dihydrofurans	256422	0.02	
class	Phenylpropanoic acid	252221	0.02	
class	Anthracenes	248470	0.02	
class	Tannins	237807	0.02	
class	Diazinanes	236042	0.02	
class	Furanoid lignans	235086	0.02	
class	Heteroaromatic comp	218542	0.02	
class	Imidazopyridines	218233	0.02	
class	Naphthofurans	195380	0.02	
class	Benzimidazoles	188111	0.02	
class	Triazinanes	182660	0.02	
class	Tetrapyrroles and de	175855	0.01	
class	Pyrrolidines	169221	0.01	
class	2-arylbenzofuran flav	147877	0.01	
class	Benzodioxoles	145446	0.01	
class	Organic carbonic acid	145287	0.01	
class	Azepines	136666	0.01	
class	Allyl sulfur compounc	125944	0.01	
class	Isoquinolines and de	113793	0.01	
class	Sulfoxides	106030	0.01	
class	Isocoumarans	103298	0.01	
class	Flavin nucleotides	99853	0.01	
class	Azaspirodecane deriv	97017	0.01	
class	Dibenzylbutane ligna	95504	0.01	
class	Aurone flavonoids	92894	0.01	
class	Diazanaphthalenes	89892	0.01	

class	Organic sulfonic acid	89671	0.01	
class	Yohimbine alkaloids	87980	0.01	
class	Biotin and derivatives	87422	0.01	
class	Dioxanes	85806	0.01	
class	Tetralins	84170	0.01	
class	Keto acids and deriva	83827	0.01	
class	Aporphines	75823	0.01	
class	Phenanthrenes and c	74874	0.01	
class	Benzothiazepines	73990	0.01	
class	Emetine alkaloids	70281	0.01	
class	Tetracyclines	65798	0.01	
class	Benzothiazoles	65048	0.01	
class	Benzazepines	63212	0.01	
class	Cinnamyl alcohols	60768	0.01	
class	Ribonucleoside 3-pho	60681	0.01	
class	Organic thiophosphoi	59596	0	
class	Pyrroles	58629	0	
class	Cinnamaldehydes	52724	0	
class	Tetrahydroisoquinolir	51498	0	
class	Benzotriazoles	50626	0	
class	Depsides and depsid	50409	0	
class	Oxanes	50116	0	
class	Lactams	48598	0	
class	Nucleoside and nucle	46980	0	
class	Saccharolipids	45144	0	
class	Ibogan-type alkaloids	44854	0	
class	Benzoxazoles	43920	0	
class	Benzodiazepines	40729	0	
class	Benzocycloheptapyri	40525	0	
class	Alkyl halides	35798	0	
class	Oxolanes	34566	0	
class	Pyrrolopyridines	34458	0	
class	(5->5)-dinucleotides	32701	0	
class	Tropane alkaloids	32269	0	
class	Indanes	31235	0	
class	Benzothiazines	30678	0	
class	Isobenzofurans	28253	0	
class	Protopine alkaloids	26350	0	
class	Azolines	23578	0	
class	Pyrazolopyridines	23001	0	
class	Furopyrans	22607	0	
class	Morphinans	21594	0	
class	Indolizidines	21351	0	

class	Ergoline and derivativ	21123	0	
class	Naphthopyrans	20890	0	
class	Benzopyrazoles	17340	0	
class	Naphthacenes	17066	0	
class	Allyl-type 1,3-dipolar	16786	0	
class	Azaphilones	16424	0	
class	Lupin alkaloids	15999	0	
class	Pyrazolo[3,4-d]pyrimi	15845	0	
class	Coumarans	15255	0	
class	Isocoumarins and de	15255	0	
class	Triazole ribonucleosic	14721	0	
class	Azolidines	14453	0	
class	Benzoxazines	13971	0	
class	Organic dithiophosph	13623	0	
class	Corynanthean-type a	13185	0	
class	Imidazole ribonucleos	12660	0	
class	Organic phosphines a	12066	0	
class	Isoindoles and deriva	11758	0	
class	Protoberberine alkalc	11495	0	
class	Phenanthrolines	10599	0	
class	Lignan lactones	10543	0	
class	Strychnos alkaloids	10207	0	
class	Azobenzenes	9454	0	
class	Sulfonyl halides	8849	0	
class	Pyrrolopyrimidine nuc	8459	0	
class	Phenol esters	8422	0	
class	Furans	8322	0	
class	Thioethers	8207	0	
class	Phthalide isoquinoline	8185	0	
class	Flavonolignans	7842	0	
class	Thioureas	7276	0	
class	Pyrimidodiazepines	6804	0	
class	Benzoxepines	6559	0	
class	Eburnan-type alkaloic	6279	0	
class	Benzothiophenes	5914	0	
class	Benzothiopyrans	5911	0	
class	Aryltetralin lignans	5244	0	
class	Cytochalasans	4959	0	
class	Plumeran-type alkalo	4799	0	
class	Angucyclines	4777	0	
class	Benzophenanthridine	4761	0	
class	Pyrrolizines	4757	0	
class	Thiadiazines	4640	0	

class	Vinca alkaloids	4637	0	
class	(3->5)-dinucleotides a	4386	0	
class	Triazolopyrimidines	4291	0	
class	Tropones	4162	0	
class	Thiophenes	4149	0	
class	Pyrrolizidines	4040	0	
class	Imidazo[1,2-a]pyrimic	3944	0	
class	Piperazinoazepines	3853	0	
class	Organic phosphonic a	3832	0	
class	Dioxolopyrans	3809	0	
class	Macrolide lactams	3619	0	
class	Benzodioxanes	3496	0	
class	Amaryllidaceae alkalo	3431	0	
class	Imidazothiazoles	3378	0	
class	Proaporphines	3166	0	
class	Thienodiazepines	2942	0	
class	Gelsemium alkaloids	2878	0	
class	Pyridopyrimidines	2516	0	
class	Pyrrolopyrimidines	2349	0	
class	Triazolopyridines	2317	0	
class	Macroline alkaloids	2306	0	
class	Furofurans	2267	0	
class	Oxazaphosphinanes	2255	0	
class	Organic disulfides	2175	0	
class	Dihydroisoquinolines	2029	0	
class	Anatoxins	2020	0	
class	Cyclobutane lignans	1815	0	
class	Vobasan alkaloids	1807	0	
class	Oxocins	1782	0	
class	Ervatamia alkaloids	1761	0	
class	Neoflavonoids	1527	0	
class	Triphenyl compounds	1478	0	
class	Thienopyrimidines	1470	0	
class	Indolonaphthyridine a	1443	0	
class	Thiosulfinic acid este	1430	0	
class	Fluorenes	1364	0	
class	Ajmaline-sarpagine a	1297	0	
class	Organic trisulfides	1259	0	
class	Alpha-methyldeoxybe	1250	0	
class	Thiadiazinanes	1233	0	
class	Pyrrolopyrazines	1142	0	
class	Furopyridines	1099	0	
class	Benzoxazepines	1095	0	

class	Endocannabinoids	1071	0	
class	Quinuclidines	1003	0	
class	Azetidines	991	0	
class	Organothiophosphoru	906	0	
class	Quinolizidines	901	0	
class	Organic thiosulfuric a	867	0	
class	Rhoeadine alkaloids	813	0	
class	Sulfamic acid derivati	743	0	
class	Isochromanequinone	712	0	
class	Camptothecins	680	0	
class	Indenes and isoinder	665	0	
class	Sulfonyls	661	0	
class	Dioxolanes	635	0	
class	Benzisoxazoles	634	0	
class	Triazolopyrazines	632	0	
class	Aristolactams	630	0	
class	Dithiolanes	627	0	
class	Thiocarbonyl compou	614	0	
class	Azepanes	599	0	
class	Imidolactams	519	0	
class	Stemona alkaloids	497	0	
class	Epoxides	491	0	
class	Diazepanes	480	0	
class	Thiochromenes	477	0	
class	Polypeptides	458	0	
class	Arylnaphthalene ligna	447	0	
class	Thiazinanes	438	0	
class	Quinodimethanes	413	0	
class	Ochratoxins and relat	412	0	
class	Homoisoflavonoids	408	0	
class	Pyridopyrazines	387	0	
class	Coumarinolignans	381	0	
class	Pyrrolines	360	0	
class	Pavine alkaloids	349	0	
class	Benzothiepins	346	0	
class	Benzothiadiazoles	340	0	
class	Aryl halides	338	0	
class	Organic oxoanionic c	326	0	
class	Pyranodioxins	323	0	
class	Perylenequinones	301	0	
class	Cephalotaxus alkaloi	299	0	
class	Sterigmatocystins	292	0	
class	Epibatidine analogue	274	0	

class	Cycloheptathiophene	249	0	
class	Loline alkaloids and c	240	0	
class	2,5-dideoxyribonucle	224	0	
class	Anthracyclines	210	0	
class	Pyrenes	165	0	
class	Benzo-1,2,3-triazines	157	0	
class	Quinolizines	156	0	
class	Aspidofractine alkaloi	144	0	
class	Thienothiazines	136	0	
class	Oxazepines	132	0	
class	Pyranopyridines	132	0	
class	Thiols	126	0	
class	6,6a-secoaporphines	124	0	
class	Sulfenyl compounds	119	0	
class	Cularin alkaloids and	117	0	
class	Aspidospermatan-typ	115	0	
class	Benzimidazole ribonu	112	0	
class	Bi- and oligothiophen	110	0	
class	Akuammilan and rela	107	0	
class	Pyrrolotriazines	101	0	
class	Benzoxadiazoles	95	0	
class	Imidazodiazepines	92	0	
class	Thienopyrans	83	0	
class	Pleiocarpaman alkalo	65	0	
class	Imidazopyrazines	60	0	
class	Pyrroloazepines	60	0	
class	Pyrazolotriazines	59	0	
class	3,4-dihydrocoumarins	54	0	
class	Metalloheterocyclic c	52	0	
class	Naphthothiazoles	49	0	
class	Quebrachamine alkal	48	0	
class	Diazepines	47	0	
class	Pyrrolopyrazoles	46	0	
class	Triazolothiazoles	43	0	
class	Unsaturated hydroca	43	0	
class	Vinylogous esters	43	0	
class	Imidazotetrazines	40	0	
class	Pyridine nucleotides	40	0	
class	Trioxolanes	38	0	
class	Triazolopyridazines	37	0	
class	Isoxazolopyridines	33	0	
class	Imidazole[4,5-c]pyridi	26	0	
class	Dioxepanes	24	0	

class	6,7-benzomorphans	23	0	
class	Isothioureas	23	0	
class	Quinolidines	20	0	
class	Furo[2,3-d]pyrimidine	18	0	
class	Pyranopyrimidines	16	0	
class	Rhazinilam alkaloids	15	0	
class	Thienopyrroles	15	0	
class	Oxazolopyridines	14	0	
class	Thiolanes	13	0	
class	Vallesaman alkaloids	13	0	
class	Imidazo[1,2-a]pyrazir	12	0	
class	Imidazo[1,2-a]pyridin	12	0	
class	Melodinus alkaloids	11	0	
class	Thienothiazoles	10	0	
class	Benzodithiins	8	0	
class	Isothiochromenes	8	0	
class	Cycloheptafurans	7	0	
class	Piperazinopiperidines	7	0	
class	Pyrazolopyrazoles	6	0	
class	Isothiocyanates	5	0	
class	Non-metal oxoanioni	4	0	
class	Isothiochromanes	3	0	
class	Brevetoxins and deri	2	0	
class	Daphniphylline-type a	2	0	
class	Erythrina alkaloids	1	0	
class	Thienoimidazoles	1	0	
class	Trithianes	1	0	

**Table S5**		
**Number of public files with organism-level metadata containing 3-hydroxy N-acyl amides**		
**Unique matches**	**Origin**	**NCBITaxonomy**
2386	Pan-ReDU	10088|Mus', '10090|Mus musculus', '10105|Mus minutoides', '10114|Rattus', '10116|Rattus norvegicus'
3010	Pan-ReDU	9606|Homo sapiens'
4278	plantMASST	10.1101/2024.05.13.593988
6505	microbeMASST	10.1038/s41564-023-01575-9
615	foodMASST	10.1038/s41538-022-00137-3

**Table S6**			
**Organ distribution in rodents by indexed spectral matches**			
UBERONBodyPartNa	n_spectral_matches	n_ion	n_compound
blood	1137	10	9
feces	401	20	19
caecum	316	8	8
ileum	144	10	10
jejunum	130	4	4
colon	52	4	4
heart	51	2	2
liver	48	6	6
duodenum	38	3	3
spleen	24	1	1
lung	20	3	3
stomach	10	1	1
upper digestive tract	6	2	2
megakaryocyte	4	1	1
missing value	4	2	2
gallbladder	1	1	1
retina	1	1	1

Table S7			
**Organ distribution in humans by indexed spectral matches**			
UBERONBodyPartNa	n_spectral_matches	n_ion	n_compound
brain	1305	9	9
feces	486	45	42
blood	270	16	16
skin	227	15	14
urine	226	12	12
milk	110	8	7
vagina	92	7	7
saliva	78	9	9
oral cavity	68	14	14
nasal cavity	49	3	3
anal region	45	2	2
epithelial cell	36	2	2
missing value	14	4	4
cerebrospinal fluid	9	3	3
alveolar system	8	4	3
bone tissue	1	1	1

Table S8					
**3-hydroxy N-acyl amide substrates used in culturing**					
Compound	Formula	MW	mg total in 1ml water	volume (if liquid at RT)	
3-Hydroxypropionic ac	C3H6O3	90.08	0.901	2.73uL (30% in water)	
3-Hydroxybutyric acid	C4H8O3	104.11	1.041	0.924uL	
3-Hydroxyvaleric acid	C5H10O3	118.13	1.181	dry	
3-Hydroxyundecanoic a	C11H22O3	202.29	2.023	2.02uL	
3-Hydroxydodecanoic a	C12H24O3	216.32	2.163	dry	
3-Hydroxytridecanoic a	C13H26O3	230.35	2.304	2.55uL	
3-Hydroxyhexadecanoi	C16H32O3	272.43	2.724	dry	

Table S9					
**MS2 mirror plots containing 3-hydroxy N-acyl amide library matches**					
**Link to metabolomics USI resolver for mirror plots for the RT experiemnts**	**Figure**	**Metabolite**	**Cosine**	**Study**	
https://metabolomics-usi.gnps2.org/dashinterface/?usi1=mzspec:GNPS2:TASK-abcfa3d422534d86b2e71a	S1(a)	3-hydroxylauroyl lysine [M+H]+	0.9082	hcom	
https://metabolomics-usi.gnps2.org/dashinterface/?usi1=mzspec:GNPS2:TASK-abcfa3d422534d86b2e71a	S1(a)	3-hydroxylauroyl 5-aminovaleric acid [M+H]+	0.9762	hcom	
https://metabolomics-usi.gnps2.org/dashinterface/?usi1=mzspec:GNPS2:TASK-abcfa3d422534d86b2e71a	S1(a)	3-hydroxylauroyl leucine [M+H]+	0.9685	hcom	
https://metabolomics-usi.gnps2.org/dashinterface/?usi1=mzspec:GNPS2:TASK-abcfa3d422534d86b2e71a	S1(a)	3-hydroxylauroyl phenylalanine [M+H]+	0.9622	hcom	
https://metabolomics-usi.gnps2.org/dashinterface/?usi1=mzspec:GNPS2:TASK-d3d7b17c76b845b6aa385	S2(a)	3-hydroxy-cis-13-octadecenoyl histamine [M+H]+	0.8863	HNRC	
https://metabolomics-usi.gnps2.org/dashinterface/?usi1=mzspec:GNPS2:TASK-9d00a020db624cc483a91	4(a)	3-hydroxytetradecanoyl glutamic acid [M+H-H2O]+	0.8149	Cadaver/Decomposition	
https://metabolomics-usi.gnps2.org/dashinterface/?usi1=mzspec%3AGNPS2%3ATASK-398fd012f8f04b8c	4(b)	3-hydroxypentanoyl leucine [M+H]+	0.6608	Osteoarthiritis	
https://metabolomics-usi.gnps2.org/dashinterface/?usi1=mzspec%3AGNPS2%3ATASK-398fd012f8f04b8c	4(c)	3-hydroxytetradecanoyl lysine [M+H]+	0.8475	Osteoarthiritis	

Table S10		
**Disease distribution in humans by indexed spectral matches**		
DOIDCommonName	n_rows	
inflammatory bowel d	159	
primary bacterial infe	120	
diabetes mellitus	60	
Alzheimer's disease	50	
obesity	40	
rheumatoid arthritis	11	
Crohn's disease	10	
chronic obstructive pu	8	
ulcerative colitis	8	
osteoarthritis	5	
Chagas disease	3	
Kawasaki disease	1	

Table S11			
**Multiplex synthesis reagents**			
**Mixture 1**	**SMILES**		
leucine	CC(C)C[C@H](N)C(O)=O		
histamine	NCCc1c[nH]cn1		
5-methoxytryptamine	COc1ccc2[nH]cc(CCN)c2c1		
tryptamine	NCCc1c[nH]c2ccccc12		
tyramine	NCCc1ccc(O)cc1		
serotonin	NCCc1c[nH]c2ccc(O)cc12		
5-aminovaleric_acid	NCCCCC(O)=O		
gamma_amino_butyr	NCCCC(O)=O		
3-methoxy tyramine	COc1cc(CCN)ccc1O		
dopamine	NCCc1ccc(O)c(O)c1		
**Mixture 2**	**SMILES**		
citrulline	N[C@@H](CCCNC(N)=O)C(O)=O		
L-2-aminoadipic acid	NC(CCCC(O)=O)C(O)=O		
Glutathione	N[C@@H](CCC(=O)N[C@@H](CS)C(=O)NCC(O)=O)C(O)=O		
ornithine	NCCC[C@H](N)C(O)=O		
homoserine	N[C@@H](CCO)C(O)=O		
(S)-2-aminopentanoic	CCC[C@H](N)C(O)=O		
propane-1,_3-diamin	NCCCN		
Putrescine	NCCCCN		
spermine	NCCCNCCCCNCCCN		
**Mixture 3**	**SMILES**		
amylamine	CCCCCN		
cadaverine	NCCCCCN		
spermidine	NCCCCNCCCN		
phenethylamine	NCCc1ccccc1		
**Mixture 4**	**SMILES**		
Valine	CC(C)[C@H](N)C(O)=O		
Tyrosine	N[C@@H](Cc1ccc(O)cc1)C(O)=O		
Tryptophan	N[C@@H](Cc1c[nH]c2ccccc12)C(O)=O		
Threonine	C[C@@H](O)[C@H](N)C(O)=O		
Serine	N[C@@H](CO)C(O)=O		
Proline	OC(=O)[C@@H]1CCCN1		
Phenylalanine	N[C@@H](Cc1ccccc1)C(O)=O		
Methionine	CSCC[C@H](N)C(O)=O		
Lysine	NCCCC[C@H](N)C(O)=O		
Histidine	N[C@@H](Cc1c[nH]cn1)C(O)=O		
Glycine	NCC(O)=O		
Glutamic_Acid	N[C@@H](CCC(O)=O)C(O)=O		
Glutamine	N[C@@H](CCC(N)=O)C(O)=O		
Cysteine	N[C@@H](CS)C(O)=O		

Aspartic Acid	N[C@@H](CC(O)=O)C(O)=O	
Asparagine	N[C@@H](CC(N)=O)C(O)=O	
Arginine	N[C@@H](CCCNC(N)=N)C(O)=O	
Alanine	C[C@H](N)C(O)=O	
isoleucine	CC[C@H](C)[C@H](N)C(O)=O	

**Table S12**	
**MZmine information**	
HNRC	https://github.com/helenamrusso/N-acyl_lipids/tree/main/Data_processing_MZmine/HIV_dataset_MSV000092833
Diabetes	https://github.com/helenamrusso/N-acyl_lipids/tree/main/Data_processing_MZmine/Re-investigation_public_datasets/Diabetes_MSV000082261
Osteoarthiritis	https://github.com/victoriadeleray/3OH_N-acyl_amides/tree/main
dead bodies	https://github.com/helenamrusso/N-acyl_lipids/tree/main/Data_processing_MZmine/Re-investigation_public_datasets/Body_decomposition_MSV000084322_MSV000084463
hcom	https://github.com/victoriadeleray/3OH_N-acyl_amides/tree/main

Table S13				
GNPS2 Molecular networking of publically downloaded data				
URL	TaskID	Name	Associated figure	MassIVE ID
https://gnps2.org/stat	d3d7b17c76b845b6a	HNRC/HIV	S2(a)	MSV000092833
https://gnps2.org/stat	33f42643a5b34d869	Diabetes	S3	MSV000082221
https://gnps2.org/stat	9d00a020db624cc48	Dead bodies/Decomp	S4(a)	MSV000084322
https://gnps2.org/stat	398fd012f8f04b8c85	Osteoarthiritis	S4(b-c)	MSV000094097

**Table S14**			
**List of all strains used in community culturing experiment**			
**Group**	**Species**		
1	Acidaminococcus_sp_D21		
1	Bacteroides_caccae_ATCC_43185		
1	Bacteroides_dorei_5_1_36/D4		
1	Bacteroides_fragilis_3_1_12		
1	Bacteroides_sp_2_1_22		
1	Catenibacterium_mitsuokai_DSM_15897		
1	Clostridium_bolteae_ATCC_BAA_613		
1	Clostridium_sp_M62-1		
1	Clostridium_sporogenes_DSM_795		
1	Collinsella_aerofaciens_ATCC_25986		
1	Coprococcus_comes_ATCC_27758		
1	Dorea_formicigenerans_ATCC_27755		
1	Dorea_longicatena_DSM_13814		
1	Enterococcus_faecalis_DSM_20478		
1	Eubacterium_rectale_ATCC_33656		
1	Mitsuokella_multacida_DSM_20544		
1	Roseburia_intestinalis_L1-82		
1	Ruminococcus_gnavus_ATCC_29149		
1	Ruminococcus_lactaris_ATCC_29176		
2	Acidaminococcus_fermentans_DSM_20731		
2	Anaerostipes_caccae_DSM_14662		
2	Anaerotruncus_colihominis_DSM_17241		
2	Bacteroides_finegoldii_DSM_17565		
2	Bacteroides_intestinalis_DSM_17393		
2	Bacteroides_ovatus_ATCC_8483		
2	Bacteroides_plebeius_DSM_17135		
2	Bacteroides_sp_1_1_6		
2	Bacteroides_sp_D2		
2	Bifidobacterium_pseudocatenulatum_DSM_20438		
2	Blautia_hansenii_DSM_20583		
2	Butyrivibrio_crossotus_DSM_2876		
2	Clostridium_hylemonae_DSM_15053		
2	Clostridium_leptum_DSM_753		
2	Clostridium_spiroforme_DSM_1552		
2	Coprococcus_eutactus_ATCC_27759		
2	Eubacterium_ventriosum_ATCC_27560		
2	Lactobacillus_plantarum_subsp_plantarum_ATCC_14917		
2	Subdoligranulum_variabile_DSM_15176		
3	Adlercreutzia_equolifaciens_DSM_19450		
3	Akkermansia_muciniphila_ATCC_BAA-835		

3	Bacteroides_stercoris_ATCC_43183	
3	Bacteroides_thetaiotaomicron_VPI_5482	
3	Bacteroides_uniformis_ATCC_8492	
3	Bifidobacterium_catenulatum_DSM_16992	
3	Blautia_hydrogenotrophica_DSM_10507	
3	Bryantella_formatexigens_DSM_14469	
3	Clostridium_scindens_ATCC_35704	
3	Clostridium_sp_L2-50	
3	Enterobacter_cloacae_subsp._cloacae_DSM_30054	
3	Eubacterium_eligens_ATCC_27750	
3	Eubacterium_siraeum_DSM_15702	
3	Faecalibacterium_prausnitzii_A2-165	
3	Holdemania_filiformis_DSM_12042	
3	Phoaeicola_dorei_DSM_17855	
3	Ruminococcus_flavefaciens_DSM_25089	
3	Ruminococcus_torques_ATCC_27756	
3	Slackia_exigua_ATCC_700122	
3	Bifidobacterium longum longum JCM 1217	
4	Alistipes_finegoldii_DSM_17242	
4	Alistipes_indistinctus_YIT_12060_DSM_22520	
4	Alistipes_onderdonkii_DSM_19147	
4	Alistipes_shahii_WAL_8301_DSM_19121	
4	Anaerofustis_stercorihominis_DSM_17244	
4	Bacteroides_coprophilus_DSM_18228	
4	Bacteroides_eggerthii_DSM_20697	
4	Bacteroides_sp_3_1_19	
4	Bacteroides_sp_9_1_42FAA	
4	Bacteroides_vulgatus_ATCC_8482	
4	Burkholderiales_bacterium_1_1_47	
4	Collinsella_stercoris_DSM_13279	
4	Desulfovibrio_piger_ATCC_29098	
4	Eubacterium_biforme_DSM_3989	
4	Eubacterium_dolichum_DSM_3991	
4	Odoribacter_splanchnicus_DSM_20712	
4	Olsenella_uli_DSM_7084	
4	Parabacteroides_distasonis_ATCC_8503	
4	Parabacteroides_merdae_ATCC_43184	
4	Phocaeicola_coprocola_DSM_17136	
4	Prevotella_copri_DSM_18205	
4	Veillonella_dispar_DSM_20735	

**Table S15**	
**Culturing media recipe**	
**hCom media**	**Quantity**
DI Water	q.s
Brain Heart Infusion Broth (RPI B11000)	35 g
Select Yeast Extract (1% w/v)	10 g
Cellobiose	2 g
D-(+)-Maltose Monohydrate	1 g
Sodium pyruvate	2 g
Proteose Peptone	5 g
Xylan (TCI X0078)	1 g
Tryptone (Difco 211921)	2.5 g
Vitamin K and Hemin Solution<br>Vitamin K – 0.5 mg/10mL<br>Hemin – 5mg/10mL	0.2 mL
Short Chain Fatty Acids Solution<br>17mL/31mL Acetic acid, glacial (Sigma A6283)<br>6mL/31mL Propionic acid (Sigma P5561)<br>4mL/31mL Butyric acid (Sigma B103500)<br>1mL/31mL Isovaleric acid (Sigma 129542)<br>1mL/31mL Iso-Butyric acid (Sigma I1754)<br>1mL/31mL n-Valeric acid (Sigma 240370)<br>1mL/31mL DL-2-methyl-Butyric acid (Sigma W269514)	3.1 mL
Trace Mineral Supplement (ATCC MD-TMS)	10 mL
Vitamin Supplement (ATCC MD-VS)	10 mL
L-Cysteine HCl (RPI C81020)	-
**Other supplements**	**Concentration (μM)**
Taurocholic acid	100
Taurochenodeoxycholic acid	100
Taurodeoxycholic acid	100
Taurolithocholic acid	100
Serotonin	100
Dopamine	100
L-DOPA	100

Table S16

GNPS2 library search results for spectral ambiguity estimation

reason_for_match	3-hydroxy N-acyl ami	GNPS compound na	SpectrumID	#Scan#	SpectrumFile	LibraryName	MQScore	TIC_Query	RT_Query	MZErrorPPM	SharedPeaks	MassDiff
not same structure	3-hydroxyhexadecan	(3-hydroxytetradecan	CCMSLIB000100130	3050	3-HYDROXY-ACYL-A	NA	0.992417	NA	NA	0	16	0
not same structure	3-hydroxypentanoyl t	N-(2-indol-3-ylethyl)(	CCMSLIB000101234	1110	3-HYDROXY-ACYL-A	NA	0.923596	NA	NA	0	6	0
not same structure	3-hydroxylauroyl 3-m	GABA-C14:1	CCMSLIB000114359	3143	3-HYDROXY-ACYL-A	NA	0.918423	NA	NA	0	6	0
not same structure	3-hydroxypropionyl gl	L-Pyroglutamic acid (	CCMSLIB000101141	132	3-HYDROXY-ACYL-A	NA	0.910596	NA	NA	0	3	0
not same structure	3-hydroxytetradecanc	Lys-C16:1	CCMSLIB000114359	3117	3-HYDROXY-ACYL-A	NA	0.901006	NA	NA	0	5	0
not same structure	3-hydroxytetradecanc	amylamine-C18:0	CCMSLIB000114355	3182	3-HYDROXY-ACYL-A	NA	0.886177	NA	NA	7.70078	5	0.00302124
not same structure	3-hydroxylauroyl phe	amylamine-C18:0	CCMSLIB000114355	3182	3-HYDROXY-ACYL-A	NA	0.886177	NA	NA	7.70078	5	0.00302124
not same structure	3-hydroxyoctadecanc	Ala-C16:1	CCMSLIB000114359	3264	3-HYDROXY-ACYL-A	NA	0.880799	NA	NA	0	10	0
not same structure	3-hydroxypentanoyl k	NCGC00386066-01!	CCMSLIB000008485	859	3-HYDROXY-ACYL-A	NA	0.845084	NA	NA	0	3	0
not same structure	3-hydroxypropionyl gl	Massbank:PB000462	CCMSLIB000002218	131	3-HYDROXY-ACYL-A	NA	0.836451	NA	NA	6.80179	4	0.00100708
not same structure	3-hydroxyhexadecan	Val-C12:1	CCMSLIB000114357	3129	3-HYDROXY-ACYL-A	NA	0.826688	NA	NA	0	4	0
not same structure	3-hydroxytetradecanc	Asp-C14:1	CCMSLIB000114358	3125	3-HYDROXY-ACYL-A	NA	0.81738	NA	NA	0	8	0
not same structure	3-hydroxydecanoyl di	Capsaicin	CCMSLIB000130355	2086	3-HYDROXY-ACYL-A	NA	0.78582	NA	NA	0	4	0
not same structure	3-hydroxyhexadecan	Ser-C16:1	CCMSLIB000114359	3217	3-HYDROXY-ACYL-A	NA	0.780178	NA	NA	0	8	0
not same structure	3-hydroxytetradecanc	Ser-C16:1	CCMSLIB000114359	3217	3-HYDROXY-ACYL-A	NA	0.780178	NA	NA	0	8	0
not same structure	3-hydroxynonanoyl G	PHENETHYLAMINE	CCMSLIB000058848	1233	3-HYDROXY-ACYL-A	NA	0.77787	NA	NA	122.84914	3	0.01499939
not same structure	3-hydroxyhexadecan	PABA-C19:0	CCMSLIB000100112	3358	3-HYDROXY-ACYL-A	NA	0.763429	NA	NA	7.14916	3	0.00299072
not same structure	3-hydroxy-cis-13-octa	PABA-C19:0	CCMSLIB000100112	3358	3-HYDROXY-ACYL-A	NA	0.763429	NA	NA	7.14916	3	0.00299072
not same structure	3-hydroxyhexadecan	Citrulline-C16:1	CCMSLIB000114359	3208	3-HYDROXY-ACYL-A	NA	0.72895	NA	NA	0	7	0
not same structure	3-hydroxypentanoyl p	N-Phenethyl-4-piperi	CCMSLIB000114295	1541	3-HYDROXY-ACYL-A	NA	0.717755	NA	NA	0	4	0
not same structure	3-hydroxypentanoyl t	4-methoxyphenylacet	CCMSLIB000174333	764	3-HYDROXY-ACYL-A	NA	0.706242	NA	NA	8.39374	3	0.0019989
same parent structure	3-hydroxylauroyl hom	(3-hydroxydodecanoy	CCMSLIB000100130	2293	3-HYDROXY-ACYL-A	NA	0.996986	NA	NA	3.16465	16	0.00100708
same parent structure	3-hydroxytetradecanc	(3-hydroxytetradecan	CCMSLIB000100130	3050	3-HYDROXY-ACYL-A	NA	0.992417	NA	NA	0	16	0
same parent structure	3-hydroxytetradecanc	(3-hydroxytetradecan	CCMSLIB000100130	3050	3-HYDROXY-ACYL-A	NA	0.992417	NA	NA	0	16	0
same parent structure	3-hydroxydecanoyl h	(3-hydroxydecanoyl)(	CCMSLIB000101294	1347	3-HYDROXY-ACYL-A	NA	0.988384	NA	NA	0	14	0
same parent structure	3-hydroxytetradecanc	N-(3-hydroxytetradec	CCMSLIB000046842	3270	3-HYDROXY-ACYL-A	NA	0.959386	NA	NA	2.56725	11	0.00100708
same parent structure	3-hydroxyhexadecan	Commendamide	CCMSLIB000046792	3236	3-HYDROXY-ACYL-A	NA	0.880246	NA	NA	0	9	0
same parent structure	3-hydroxytetradecanc	cadaverine_3-hydrox	CCMSLIB000162115	2666	3-HYDROXY-ACYL-A	NA	0.755797	NA	NA	2.96542	9	9.77E-04
same ion	3-hydroxytetradecanc	histamine-C14:1	CCMSLIB000114359	2605	3-HYDROXY-ACYL-A	NA	0.947406	NA	NA	0	5	0
same ion	3-hydroxylauroyl hist	histamine-C12:1	CCMSLIB000114358	1705	3-HYDROXY-ACYL-A	NA	0.944492	NA	NA	0	5	0
same ion	3-hydroxyhexadecan	histamine-C16:1	CCMSLIB000114360	3092	3-HYDROXY-ACYL-A	NA	0.918548	NA	NA	2.89141	6	0.00100708
same ion	3-hydroxytetradecanc	GABA-C14:1	CCMSLIB000114359	3143	3-HYDROXY-ACYL-A	NA	0.918423	NA	NA	0	6	0
same ion	3-hydroxyhexadecan	(Z)-N-hexadec-9-eno	CCMSLIB000046842	3394	3-HYDROXY-ACYL-A	NA	0.911824	NA	NA	2.5033	7	0.00100708
same ion	3-hydroxytetradecanc	amylamine-C14:1	CCMSLIB000114359	3309	3-HYDROXY-ACYL-A	NA	0.911455	NA	NA	0	12	0
same ion	3-hydroxyhexadecan	Lys-C16:1	CCMSLIB000114359	3117	3-HYDROXY-ACYL-A	NA	0.901006	NA	NA	0	5	0
same ion	3-hydroxylauroyl orni	Ornithine-C12:1	CCMSLIB000114357	1719	3-HYDROXY-ACYL-A	NA	0.889743	NA	NA	0	9	0
same ion	3-hydroxylauroyl GAE	GABA-C12:1	CCMSLIB000114357	2668	3-HYDROXY-ACYL-A	NA	0.88919	NA	NA	0	5	0
same ion	3-hydroxyhexadecan	Tyr-C16:1	CCMSLIB000100108	3252	3-HYDROXY-ACYL-A	NA	0.884648	NA	NA	0	9	0
same ion	3-hydroxyhexadecan	Ala-C16:1	CCMSLIB000114359	3264	3-HYDROXY-ACYL-A	NA	0.880799	NA	NA	0	10	0
same ion	3-hydroxylauroyl histi	His-C12:1	CCMSLIB000100114	1625	3-HYDROXY-ACYL-A	NA	0.877727	NA	NA	0	6	0
same ion	3-hydroxyundecanoyl	amylamine-C11:1	CCMSLIB000114357	3212	3-HYDROXY-ACYL-A	NA	0.849306	NA	NA	0	9	0
same ion	3-hydroxylauroyl vali	Val-C12:1	CCMSLIB000114357	3129	3-HYDROXY-ACYL-A	NA	0.826688	NA	NA	0	4	0
same ion	3-hydroxylauroyl thre	Thr-C12:1	CCMSLIB000114357	2435	3-HYDROXY-ACYL-A	NA	0.81999	NA	NA	0	8	0
same ion	3-hydroxylauroyl glut	Gln-C12:1	CCMSLIB000114357	2156	3-HYDROXY-ACYL-A	NA	0.815472	NA	NA	0	8	0
same ion	3-hydroxytetradecanc	Gly-C14:1	CCMSLIB000114359	3141	3-HYDROXY-ACYL-A	NA	0.808108	NA	NA	0	8	0
same ion	3-hydroxylauroyl alan	Ala-C12:1	CCMSLIB000114357	2677	3-HYDROXY-ACYL-A	NA	0.791227	NA	NA	0	8	0
same ion	3-hydroxyhexadecan	Ser-C16:1	CCMSLIB000114359	3217	3-HYDROXY-ACYL-A	NA	0.780178	NA	NA	0	8	0
same ion	3-hydroxylauroyl met	Met-C12:1	CCMSLIB000114357	3111	3-HYDROXY-ACYL-A	NA	0.763702	NA	NA	0	9	0
same ion	3-hydroxylauroyl glyc	Gly-C12:1	CCMSLIB000114357	2500	3-HYDROXY-ACYL-A	NA	0.76268	NA	NA	0	8	0
same ion	3-hydroxylauroyl putr	putrescine-C12:1	CCMSLIB000114358	1685	3-HYDROXY-ACYL-A	NA	0.760646	NA	NA	0	8	0
same ion	3-hydroxylauroyl asp	Asn-C12:1	CCMSLIB000114357	2108	3-HYDROXY-ACYL-A	NA	0.760503	NA	NA	0	9	0
same ion	3-hydroxyhexadecan	Citrulline-C16:1	CCMSLIB000114359	3208	3-HYDROXY-ACYL-A	NA	0.72895	NA	NA	0	7	0
same ion	3-hydroxy-cis-13-octa	amylamine-C18:0	CCMSLIB000114355	3182	3-HYDROXY-ACYL-A	NA	0.886177	NA	NA	7.70078	5	0.00302124
structural isomer	3-hydroxylauroyl thre	(3-hydroxydodecanoy	CCMSLIB000100130	2424	3-HYDROXY-ACYL-A	NA	0.978428	NA	NA	0	13	0
structural isomer	3-hydroxydecanoyl th	(3-hydroxydecanoyl)(	CCMSLIB000101294	1520	3-HYDROXY-ACYL-A	NA	0.966034	NA	NA	0	10	0
structural isomer	3-hydroxyhexadecan	(3-hydroxytetradecan	CCMSLIB000100130	3127	3-HYDROXY-ACYL-A	NA	0.950726	NA	NA	2.90846	13	0.00100708
structural isomer	3-hydroxytetradecanc	(3-hydroxytetradecan	CCMSLIB000100130	3127	3-HYDROXY-ACYL-A	NA	0.950726	NA	NA	2.90846	13	0.00100708
structural isomer	3-hydroxydecanoyl s	10-hydroxydecanoic-(	CCMSLIB000174330	2337	3-HYDROXY-ACYL-A	NA	0.849517	NA	NA	11.51318	8	0.0039978
structural isomer	3-hydroxylauroyl hom	OH-C12-HSL	CCMSLIB000054636	2295	3-HYDROXY-ACYL-A	NA	0.795239	NA	NA	0	9	0
structural isomer	3-hydroxypentanoyl s	(3-hydroxybutanoyl)h	CCMSLIB000100130	333	3-HYDROXY-ACYL-A	NA	0.733329	NA	NA	4.81228	5	9.92E-04
structural isomer	3-hydroxydecanoyl 3-	10-hydroxydecanoic-(	CCMSLIB000174330	2557	3-HYDROXY-ACYL-A	NA	0.709508	NA	NA	14.79697	4	0.00500488
structural isomer	3-hydroxypentanoyl t	cyclobutanecarboxyli	CCMSLIB000162120	1114	3-HYDROXY-ACYL-A	NA	0.788153	NA	NA	4.07905	3	9.92E-04

reason_for_match	SpecMZ	SpecCharge	FileScanUniqueID	NumberHits	Ion_Source	Instrument	Compound_Source	PI	Data_Collector	Adduct	Precursor_MZ	ExactMass	Charge	CAS_Number	Pubmed_ID
not same structure	346.259	1	3-HYDROXY-ACYL-A	1	LC-ESI	Orbitrap	Lysate	RODRIGUES	RODRIGUES	M+H	346.259	345.251	1	NA	NA
not same structure	261.16	1	3-HYDROXY-ACYL-A	1	LC-ESI	Orbitrap	Commercial	Trent Northen	JGI	M+H	261.16	0	1	N/A	N/A
not same structure	312.253	1	3-HYDROXY-ACYL-A	1	ESI	Orbitrap	Commercial	Dorrestein	Ipsita	M+H	312.253	311.246	1	N/A	N/A
not same structure	130.05	1	3-HYDROXY-ACYL-A	1	LC-ESI	Orbitrap	Commercial	Trent Northen	JGI	M+H	130.05	0	1	N/A	N/A
not same structure	383.327	1	3-HYDROXY-ACYL-A	1	ESI	Orbitrap	Commercial	Dorrestein	Ipsita	M+H	383.327	382.32	1	N/A	N/A
not same structure	392.326	1	3-HYDROXY-ACYL-A	1	ESI	Orbitrap	Commercial	Dorrestein	Ipsita	M+K	392.329	353.366	1	N/A	N/A
not same structure	392.326	1	3-HYDROXY-ACYL-A	1	ESI	Orbitrap	Commercial	Dorrestein	Ipsita	M+K	392.329	353.366	1	N/A	N/A
not same structure	326.269	1	3-HYDROXY-ACYL-A	1	ESI	Orbitrap	Commercial	Dorrestein	Ipsita	M+H	326.269	325.262	1	N/A	N/A
not same structure	232.154	1	3-HYDROXY-ACYL-A	1	LC-ESI	Maxis II HD Q-TOF B	isolated	Jadhav/Dorrestein	lfnothias	M+H	232.154	231.147	1	n/a	n/a
not same structure	148.06	1	3-HYDROXY-ACYL-A	1	ESI	LC-ESI-QTOF	Isolated	Putative Massbank M	Massbank	[M+H]+	148.061	0	1	N/A	N/A
not same structure	298.238	1	3-HYDROXY-ACYL-A	1	ESI	Orbitrap	Commercial	Dorrestein	Ipsita	M+H	298.238	297.23	1	N/A	N/A
not same structure	356.243	1	3-HYDROXY-ACYL-A	1	ESI	Orbitrap	Commercial	Dorrestein	Ipsita	M+H	356.243	355.236	1	N/A	N/A
not same structure	306.206	1	3-HYDROXY-ACYL-A	1	ESI	Orbitrap	Commercial	Tomas Pluskal	Corinna Brungs	[M+H]+	306.206	305.199	1	N/A	N/A
not same structure	342.264	1	3-HYDROXY-ACYL-A	1	ESI	Orbitrap	Commercial	Dorrestein	Ipsita	M+H	342.264	341.257	1	N/A	N/A
not same structure	342.264	1	3-HYDROXY-ACYL-A	1	ESI	Orbitrap	Commercial	Dorrestein	Ipsita	M+H	342.264	341.257	1	N/A	N/A
not same structure	122.081	1	3-HYDROXY-ACYL-A	1	ESI	Orbitrap	Isolated	Madeleine Ernst	Anna Abrahamsson	M+H	122.096	0	1	64-04-0	N/A
not same structure	418.329	1	3-HYDROXY-ACYL-A	1	ESI	qTof	Crude	Dorrestein	Emily Gentry	M+H	418.332	417.324	1	N/A	N/A
not same structure	418.329	1	3-HYDROXY-ACYL-A	1	ESI	qTof	Crude	Dorrestein	Emily Gentry	M+H	418.332	417.324	1	N/A	N/A
not same structure	412.317	1	3-HYDROXY-ACYL-A	1	ESI	Orbitrap	Commercial	Dorrestein	Ipsita	M+H	412.317	411.31	1	N/A	N/A
not same structure	204.138	1	3-HYDROXY-ACYL-A	1	LC-ESI	qToF	Commercial	Barkholtz	Pieters	M+H	204.138	204.138	1	39742-60-4	96437
not same structure	238.144	1	3-HYDROXY-ACYL-A	1	LC-ESI	ToF	Crude	Pieter Dorrestein	victoriadeleray	[M+H]+	238.142	237.135	1	N/A	N/A
same parent structure	318.227	1	3-HYDROXY-ACYL-A	1	LC-ESI	Orbitrap	Lysate	RODRIGUES	RODRIGUES	M+H	318.228	317.22	1	NA	NA
same parent structure	346.259	1	3-HYDROXY-ACYL-A	1	LC-ESI	Orbitrap	Lysate	RODRIGUES	RODRIGUES	M+H	346.259	345.251	1	NA	NA
same parent structure	346.259	1	3-HYDROXY-ACYL-A	1	LC-ESI	Orbitrap	Lysate	RODRIGUES	RODRIGUES	M+H	346.259	345.251	1	NA	NA
same parent structure	290.196	1	3-HYDROXY-ACYL-A	1	LC-ESI	Orbitrap	Lysate	RODRIGUES	RODRIGUES	M+H	290.196	0	1	NA	NA
same parent structure	392.279	1	3-HYDROXY-ACYL-A	1	LC-ESI	Orbitrap	Isolated	Veronique EPARVIEF	Leonie PELLISSIER	M+H	392.28	391.272	1	NA	NA
same parent structure	330.264	1	3-HYDROXY-ACYL-A	1	LC-ESI	qTof	Lysate	Dorrestein	aaksenov	M+H	330.264	0	0	NA	NA
same parent structure	329.316	1	3-HYDROXY-ACYL-A	1	LC-ESI	ToF	Crude	Pieter Dorrestein	victoriadeleray	[M+H]+	329.317	328.309	1	NA	NA
same ion	320.27	1	3-HYDROXY-ACYL-A	1	ESI	Orbitrap	Commercial	Dorrestein	Ipsita	M+H	320.27	319.262	1	N/A	N/A
same ion	292.238	1	3-HYDROXY-ACYL-A	1	ESI	Orbitrap	Commercial	Dorrestein	Ipsita	M+H	292.238	291.231	1	N/A	N/A
same ion	348.3	1	3-HYDROXY-ACYL-A	1	ESI	Orbitrap	Commercial	Dorrestein	Ipsita	M+H	348.301	347.294	1	N/A	N/A
same ion	312.253	1	3-HYDROXY-ACYL-A	1	ESI	Orbitrap	Commercial	Dorrestein	Ipsita	M+H	312.253	311.246	1	N/A	N/A
same ion	402.3	1	3-HYDROXY-ACYL-A	1	LC-ESI	Orbitrap	Isolated	Veronique EPARVIEF	Leonie PELLISSIER	M+H	402.301	401.293	1	NA	NA
same ion	296.295	1	3-HYDROXY-ACYL-A	1	ESI	Orbitrap	Commercial	Dorrestein	Ipsita	M+H	296.295	295.288	1	N/A	N/A
same ion	383.327	1	3-HYDROXY-ACYL-A	1	ESI	Orbitrap	Commercial	Dorrestein	Ipsita	M+H	383.327	382.32	1	N/A	N/A
same ion	313.249	1	3-HYDROXY-ACYL-A	1	ESI	Orbitrap	Commercial	Dorrestein	Ipsita	M+H	313.249	312.241	1	N/A	N/A
same ion	284.222	1	3-HYDROXY-ACYL-A	1	ESI	Orbitrap	Commercial	Dorrestein	Ipsita	M+H	284.222	283.215	1	N/A	N/A
same ion	418.295	1	3-HYDROXY-ACYL-A	1	ESI	qTof	Crude	Dorrestein	Emily Gentry	M+H	418.295	417.288	1	N/A	N/A
same ion	326.269	1	3-HYDROXY-ACYL-A	1	ESI	Orbitrap	Commercial	Dorrestein	Ipsita	M+H	326.269	325.262	1	N/A	N/A
same ion	336.228	1	3-HYDROXY-ACYL-A	1	ESI	qTof	Crude	Dorrestein	Emily Gentry	M+H	336.228	335.221	1	N/A	N/A
same ion	254.248	1	3-HYDROXY-ACYL-A	1	ESI	Orbitrap	Commercial	Dorrestein	Ipsita	M+H	254.248	253.241	1	N/A	N/A
same ion	298.238	1	3-HYDROXY-ACYL-A	1	ESI	Orbitrap	Commercial	Dorrestein	Ipsita	M+H	298.238	297.23	1	N/A	N/A
same ion	300.217	1	3-HYDROXY-ACYL-A	1	ESI	Orbitrap	Commercial	Dorrestein	Ipsita	M+H	300.217	299.21	1	N/A	N/A
same ion	327.228	1	3-HYDROXY-ACYL-A	1	ESI	Orbitrap	Commercial	Dorrestein	Ipsita	M+H	327.228	326.221	1	N/A	N/A
same ion	284.222	1	3-HYDROXY-ACYL-A	1	ESI	Orbitrap	Commercial	Dorrestein	Ipsita	M+H	284.222	283.215	1	N/A	N/A
same ion	270.206	1	3-HYDROXY-ACYL-A	1	ESI	Orbitrap	Commercial	Dorrestein	Ipsita	M+H	270.206	269.199	1	N/A	N/A
same ion	342.264	1	3-HYDROXY-ACYL-A	1	ESI	Orbitrap	Commercial	Dorrestein	Ipsita	M+H	342.264	341.257	1	N/A	N/A
same ion	330.21	1	3-HYDROXY-ACYL-A	1	ESI	Orbitrap	Commercial	Dorrestein	Ipsita	M+H	330.21	329.202	1	N/A	N/A
same ion	256.191	1	3-HYDROXY-ACYL-A	1	ESI	Orbitrap	Commercial	Dorrestein	Ipsita	M+H	256.191	255.183	1	N/A	N/A
same ion	269.259	1	3-HYDROXY-ACYL-A	1	ESI	Orbitrap	Commercial	Dorrestein	Ipsita	M+H	269.259	268.251	1	N/A	N/A
same ion	313.212	1	3-HYDROXY-ACYL-A	1	ESI	Orbitrap	Commercial	Dorrestein	Ipsita	M+H	313.212	312.205	1	N/A	N/A
same ion	412.317	1	3-HYDROXY-ACYL-A	1	ESI	Orbitrap	Commercial	Dorrestein	Ipsita	M+H	412.317	411.31	1	N/A	N/A
same ion	392.326	1	3-HYDROXY-ACYL-A	1	ESI	Orbitrap	Commercial	Dorrestein	Ipsita	M+K	392.329	353.366	1	N/A	N/A
structural isomer	318.228	1	3-HYDROXY-ACYL-A	1	LC-ESI	Orbitrap	Lysate	RODRIGUES	RODRIGUES	M+H	318.228	317.22	1	NA	NA
structural isomer	290.196	1	3-HYDROXY-ACYL-A	1	LC-ESI	Orbitrap	Lysate	RODRIGUES	RODRIGUES	M+H	290.196	0	1	NA	NA
structural isomer	346.258	1	3-HYDROXY-ACYL-A	1	LC-ESI	Orbitrap	Lysate	RODRIGUES	RODRIGUES	M+H	346.259	345.251	1	NA	NA
structural isomer	346.258	1	3-HYDROXY-ACYL-A	1	LC-ESI	Orbitrap	Lysate	RODRIGUES	RODRIGUES	M+H	346.259	345.251	1	NA	NA
structural isomer	347.233	1	3-HYDROXY-ACYL-A	1	LC-ESI	ToF	Crude	Pieter Dorrestein	victoriadeleray	[M+H]+	347.237	346.23	1	N/A	N/A
structural isomer	300.217	0	3-HYDROXY-ACYL-A	1	LC-ESI	Orbitrap	Commercial	Rodrigues	Rodrigues	M+H	300.217	0	0	NA	NA
structural isomer	206.103	1	3-HYDROXY-ACYL-A	1	LC-ESI	Orbitrap	Other	Alice Rodrigues	Alice Rodrigues	M+H	206.102	205.095	1	NA	NA
structural isomer	338.232	1	3-HYDROXY-ACYL-A	1	LC-ESI	ToF	Crude	Pieter Dorrestein	victoriadeleray	[M+H]+	338.237	337.23	1	N/A	N/A
structural isomer	243.149	1	3-HYDROXY-ACYL-A	1	LC-ESI	ToF	Crude	Pieter Dorrestein	victoriadeleray	[M+H]+	243.15	242.142	1	N/A	N/A

reason_for_match	Smiles	INCHI	INCHI_AUX	Library_Class	IonMode	Organism	LibMZ	UpdateWorkflowNam	LibraryQualityString	tags	molecular_formula	InChIKey	InChIKey-Planar	superclass	class	subclass	npclassifier_supercla	npclassifier_class	npclassifier_pathway	library_usi
not same structure	O=C(O)C(NC(CC(O)	InChI=1S/C18H35NC	NA	3	Positive	GNPS-LIBRARY	346.259	UPDATE-SINGLE-A	Bronze	NA	C18H35NO5	YKQKOGLJZIAXBT-I	YKQKOGLJZIAXBT	Organic acids and de	Carboxylic acids and	Amino acids, peptide	Fatty amides	N-acyl amines	Fatty acids	mzspec:GNPS:GNPS
not same structure	CC(C)COC(O)=NCC(	InChI=1S/C15H20N2	N/A	3	Positive	BERKELEY-LAB	261.16	UPDATE-SINGLE-A	Bronze	NA	C15H20N2O2	XYRFMAZHKFPQBJ	XYRFMAZHKFPQBJ	Organoheterocyclic c	Indoles and derivative	Indoles	Tryptophan alkaloids	Simple indole alkaloic	Alkaloids	mzspec:GNPS:GNPS
not same structure	O=C(NCCCC(O)=O)(	InChI=1S/C18H33NC	N/A	1	Positive	ECG-ACYL-AMIDES	312.253	UPDATE-SINGLE-A	Gold	NA	C18H33NO3	FWCRRKCWDXNAS	FWCRRKCWDXNAS	Lipids and lipid-like m	Fatty Acyls	Fatty amides	Fatty amides	N-acyl amines	Fatty acids	mzspec:GNPS:GNPS
not same structure	O=C(O)C1CCC(O)=N	InChI=1S/C5H7NO3/	N/A	3	Positive	BERKELEY-LAB	130.05	UPDATE-SINGLE-A	Bronze	NA	C5H7NO3	ODHCTXKNWHHXJ	ODHCTXKNWHHXJ	Organic acids and de	Carboxylic acids and	Amino acids, peptide	Small peptides	Aminoacids	Amino acids and Pep	mzspec:GNPS:GNPS
not same structure	O=C(NC(CCCCN)C(	InChI=1S/C22H42N2	N/A	1	Positive	ECG-ACYL-AMIDES	383.327	UPDATE-SINGLE-A	Gold	NA	C22H42N2O3	ZHRUVKURTCPCET	ZHRUVKURTCPCET	Organic acids and de	Carboxylic acids and	Amino acids, peptide	Fatty amides	N-acyl amines	Fatty acids	mzspec:GNPS:GNPS
not same structure	CCCCCCCCCCCCC	InChI=1S/C23H47NC	N/A	1	Positive	ECG-ACYL-AMIDES	392.329	UPDATE-SINGLE-A	Gold	NA	C23H47NO	BNMNWSJQGWRN4	BNMNWSJQGWRN4	Lipids and lipid-like m	Fatty Acyls	Fatty amides	Fatty amides	N-acyl amines	Fatty acids	mzspec:GNPS:GNPS
not same structure	CCCCCCCCCCCCC	InChI=1S/C23H47NC	N/A	1	Positive	ECG-ACYL-AMIDES	392.329	UPDATE-SINGLE-A	Gold	NA	C23H47NO	BNMNWSJQGWRN4	BNMNWSJQGWRN4	Lipids and lipid-like m	Fatty Acyls	Fatty amides	Fatty amides	N-acyl amines	Fatty acids	mzspec:GNPS:GNPS
not same structure	O=C(NC(C(O)=O)C(	InChI=1S/C19H35NC	N/A	1	Positive	ECG-ACYL-AMIDES	326.269	UPDATE-SINGLE-A	Gold	NA	C19H35NO3	FIWFAXHHHSXJMC	FIWFAXHHHSXJMC	Organic acids and de	Carboxylic acids and	Amino acids, peptide	Fatty amides	N-acyl amines	Fatty acids	mzspec:GNPS:GNPS
not same structure	CC(C)CC(NC(=O)C(	InChI=1S/C11H21NC	N/A	1	positive	GNPS-NIH-NATURA	232.154	UPDATE-SINGLE-A	Gold	NA	C11H21NO4	SWEPQJJUAVEABL	SWEPQJJUAVEABL	N/A	N/A	N/A	Small peptides	Dipeptides	Amino acids and Pep	mzspec:GNPS:GNPS
not same structure	C(CC(=O)O)C(C(=O)	1S/C5H9NO4/c6-3(5(	N/A	3	Positive	MASSBANK	148.061	UPDATE-SINGLE-A	Bronze	NA	C5H9NO4	WHUUTDBJXJRKM	WHUUTDBJXJRKM	Organic acids and de	Carboxylic acids and	Amino acids, peptide	Small peptides	Aminoacids	Amino acids and Pep	mzspec:GNPS:GNPS
not same structure	O=C(NC(C(O)=O)C(	InChI=1S/C17H31NC	N/A	1	Positive	ECG-ACYL-AMIDES	298.238	UPDATE-SINGLE-A	Gold	NA	C17H31NO3	GIBJNGPXLZPUCZ-	GIBJNGPXLZPUCZ	Organic acids and de	Carboxylic acids and	Amino acids, peptide	Fatty amides	N-acyl amines	Fatty acids	mzspec:GNPS:GNPS
not same structure	O=C(NC(C(O)=O)CC	InChI=1S/C19H33NC	N/A	1	Positive	ECG-ACYL-AMIDES	356.243	UPDATE-SINGLE-A	Gold	NA	C19H33NO5	WNWJRHRYMQWTI	WNWJRHRYMQWTI	Organic acids and de	Carboxylic acids and	Amino acids, peptide	Fatty amides	N-acyl amines	Fatty acids	mzspec:GNPS:GNPS
not same structure	COc1c(O)ccc(CNC(=	InChI=1S/C18H27NC	YKPUWZUDDOIDPN	1	Positive	MSNLIB-POSITIVE	306.206	UPDATE-SINGLE-A	Gold	NA	C18H27NO3	YKPUWZUDDOIDPN	YKPUWZUDDOIDPN	Benzenoids	Phenols	Methoxyphenols	Pseudoalkaloids	Capsaicins and Caps	Alkaloids	mzspec:GNPS:GNPS
not same structure	O=C(NC(C(O)=O)C(	InChI=1S/C19H35NC	N/A	1	Positive	ECG-ACYL-AMIDES	342.264	UPDATE-SINGLE-A	Gold	NA	C19H35NO4	UISJHGBYJQEQBK-	UISJHGBYJQEQBK	Organic acids and de	Carboxylic acids and	Amino acids, peptide	Fatty amides	N-acyl amines	Fatty acids	mzspec:GNPS:GNPS
not same structure	O=C(NC(C(O)=O)C(	InChI=1S/C19H35NC	N/A	1	Positive	ECG-ACYL-AMIDES	342.264	UPDATE-SINGLE-A	Gold	NA	C19H35NO4	UISJHGBYJQEQBK-	UISJHGBYJQEQBK	Organic acids and de	Carboxylic acids and	Amino acids, peptide	Fatty amides	N-acyl amines	Fatty acids	mzspec:GNPS:GNPS
not same structure	NCCC1=CC=CC=C1	InChI=1S/C8H11N/c	N/A	1	Positive	GNPS-LIBRARY	122.096	UPDATE-SINGLE-A	Gold	NA	C8H11N	BHHGXPLMPWCGH	BHHGXPLMPWCGH	Benzenoids	Benzene and substitu	Phenethylamines	Tyrosine alkaloids	Phenylethylamines	Alkaloids	mzspec:GNPS:GNPS
not same structure	CCCCCCCCCCCCC	InChI=1S/C26H43NC	N/A	2	Positive	ECG-ACYL-AMIDES	418.332	UPDATE-SINGLE-A	Silver	NA	C26H43NO3	FMEWBLCGEAGUF	FMEWBLCGEAGUF	Benzenoids	Benzene and substitu	Benzoic acids and de	Anthranilic acid alkalc	Anthranilic acid deriv	Alkaloids	mzspec:GNPS:GNPS
not same structure	CCCCCCCCCCCCC	InChI=1S/C26H43NC	N/A	2	Positive	ECG-ACYL-AMIDES	418.332	UPDATE-SINGLE-A	Silver	NA	C26H43NO3	FMEWBLCGEAGUF	FMEWBLCGEAGUF	Benzenoids	Benzene and substitu	Benzoic acids and de	Anthranilic acid alkalc	Anthranilic acid deriv	Alkaloids	mzspec:GNPS:GNPS
not same structure	O=C(NC(C(O)=O)CC	InChI=1S/C22H41N3	N/A	1	Positive	ECG-ACYL-AMIDES	412.317	UPDATE-SINGLE-A	Gold	NA	C22H41N3O4	HLGPQXBQDRNSH	HLGPQXBQDRNSH	Organic acids and de	Carboxylic acids and	Amino acids, peptide	Fatty amides	N-acyl amines	Fatty acids	mzspec:GNPS:GNPS
not same structure	C1CN(CCC1=O)CC(	1S/C13H17NO/c15-1	N/A	2	Positive	DRUGS-OF-ABUSE-	204.138	UPDATE-SINGLE-A	Silver	NA	C13H17NO	YDJXNYNKKXZBMP	YDJXNYNKKXZBMP	Benzenoids	Benzene and substitu	Phenethylamines	Lysine alkaloids	Piperidine alkaloids	Alkaloids	mzspec:GNPS:GNPS
not same structure	COc1ccc(CC(=O)OC	InChI=1S/C13H19NC	N/A	1	Positive	GNPS-ION-MOBILIT	238.142	UPDATE-SINGLE-A	Gold	NA	C13H19NO3	GVYPOLOJPZSWN	GVYPOLOJPZSWN	N/A	N/A	N/A	NA	NA	NA	mzspec:GNPS:GNPS
same parent structure	O=C(O)C(NC(CC(O)	InChI=1S/C16H31NC	NA	3	Positive	GNPS-LIBRARY	318.228	UPDATE-SINGLE-A	Bronze	NA	C16H31NO5	MYAXJEBANUBRM	MYAXJEBANUBRM	Organic acids and de	Carboxylic acids and	Amino acids, peptide	Fatty amides	N-acyl amines	Fatty acids	mzspec:GNPS:GNPS
same parent structure	O=C(O)C(NC(CC(O)	InChI=1S/C18H35NC	NA	3	Positive	GNPS-LIBRARY	346.259	UPDATE-SINGLE-A	Bronze	NA	C18H35NO5	YKQKOGLJZIAXBT-I	YKQKOGLJZIAXBT	Organic acids and de	Carboxylic acids and	Amino acids, peptide	Fatty amides	N-acyl amines	Fatty acids	mzspec:GNPS:GNPS
same parent structure	O=C(O)C(NC(CC(O)	InChI=1S/C18H35NC	NA	3	Positive	GNPS-LIBRARY	346.259	UPDATE-SINGLE-A	Bronze	NA	C18H35NO5	YKQKOGLJZIAXBT-I	YKQKOGLJZIAXBT	Organic acids and de	Carboxylic acids and	Amino acids, peptide	Fatty amides	N-acyl amines	Fatty acids	mzspec:GNPS:GNPS
same parent structure	NA	NA	NA	3	Positive	GNPS-LIBRARY	290.196	UPDATE-SINGLE-A	Bronze	NA	N/A	N/A	N/A	N/A	N/A	N/A	N/A	N/A	N/A	mzspec:GNPS:GNPS
same parent structure	CCCCCCCCCCCC(	InChI=1S/C23H37NC	NA	3	Positive	GNPS-LIBRARY	392.28	UPDATE-SINGLE-A	Bronze	NA	C23H37NO4	CSDAKHXEYAPDJI-	CSDAKHXEYAPDJI	Organic acids and de	Carboxylic acids and	Amino acids, peptide	Fatty amides	N-acyl amines	Fatty acids	mzspec:GNPS:GNPS
same parent structure	NA	NA	NA	3	Positive	GNPS-LIBRARY	330.264	UPDATE-SINGLE-A	Bronze	NA	N/A	N/A	N/A	N/A	N/A	N/A	N/A	N/A	N/A	mzspec:GNPS:GNPS
same parent structure	CCCCCCCCCCCC(	InChI=1S/C19H40N2	NA	1	Positive	GNPS-ION-MOBILIT	329.317	UPDATE-SINGLE-A	Gold	NA	C19H40N2O2	DYAMKNXBGAAFTI-	DYAMKNXBGAAFTI	N/A	N/A	N/A	Fatty amides	N-acyl amines	Fatty acids	mzspec:GNPS:GNPS
same ion	O=C(NCCC1=NC=C	InChI=1S/C19H33N3	N/A	1	Positive	ECG-ACYL-AMIDES	320.27	UPDATE-SINGLE-A	Gold	NA	C19H33N3O	ZZTCBWSNGKFSTC	ZZTCBWSNGKFSTC	Lipids and lipid-like m	Fatty Acyls	Fatty amides	Fatty amides	N-acyl amines	Fatty acids	mzspec:GNPS:GNPS
same ion	O=C(NCCC1=NC=C	InChI=1S/C17H29N3	N/A	1	Positive	ECG-ACYL-AMIDES	292.238	UPDATE-SINGLE-A	Gold	NA	C17H29N3O	FCAVSASQOOTNDN	FCAVSASQOOTNDN	Lipids and lipid-like m	Fatty Acyls	Fatty amides	Fatty amides	N-acyl amines	Fatty acids	mzspec:GNPS:GNPS
same ion	O=C(NCCC1=NC=C	InChI=1S/C21H37N3	N/A	1	Positive	ECG-ACYL-AMIDES	348.301	UPDATE-SINGLE-A	Gold	NA	C21H37N3O	VKCRNDVFUWCOD	VKCRNDVFUWCOD	Lipids and lipid-like m	Fatty Acyls	Fatty amides	Fatty amides	N-acyl amines	Fatty acids	mzspec:GNPS:GNPS
same ion	O=C(NCCCC(O)=O)(	InChI=1S/C18H33NC	N/A	1	Positive	ECG-ACYL-AMIDES	312.253	UPDATE-SINGLE-A	Gold	NA	C18H33NO3	FWCRRKCWDXNAS	FWCRRKCWDXNAS	Lipids and lipid-like m	Fatty Acyls	Fatty amides	Fatty amides	N-acyl amines	Fatty acids	mzspec:GNPS:GNPS
same ion	O=C(N[C@@H](CC1	InChI=1S/C25H39NC	NA	3	Positive	GNPS-LIBRARY	402.301	UPDATE-SINGLE-A	Bronze	NA	C25H39NO3	OIDVFUQQVKYQTG	OIDVFUQQVKYQTG	Organic acids and de	Carboxylic acids and	Amino acids, peptide	Fatty amides	N-acyl amines	Fatty acids	mzspec:GNPS:GNPS
same ion	O=C(NCCCCC)CCO	InChI=1S/C19H37NC	N/A	1	Positive	ECG-ACYL-AMIDES	296.295	UPDATE-SINGLE-A	Gold	NA	C19H37NO	YFMJPCYNBKAZFK	YFMJPCYNBKAZFK	Lipids and lipid-like m	Fatty Acyls	Fatty amides	Fatty amides	N-acyl amines	Fatty acids	mzspec:GNPS:GNPS
same ion	O=C(NC(CCCCN)C(	InChI=1S/C22H42N2	N/A	1	Positive	ECG-ACYL-AMIDES	383.327	UPDATE-SINGLE-A	Gold	NA	C22H42N2O3	ZHRUVKURTCPCET	ZHRUVKURTCPCET	Organic acids and de	Carboxylic acids and	Amino acids, peptide	Fatty amides	N-acyl amines	Fatty acids	mzspec:GNPS:GNPS
same ion	O=C(NC(CCCN)C(O	InChI=1S/C17H32N2	N/A	1	Positive	ECG-ACYL-AMIDES	313.249	UPDATE-SINGLE-A	Gold	NA	C17H32N2O3	MDJFAFPATBTHAD-	MDJFAFPATBTHAD	Organic acids and de	Carboxylic acids and	Amino acids, peptide	Fatty amides	N-acyl amines	Fatty acids	mzspec:GNPS:GNPS
same ion	O=C(NCCCC(O)=O)(	InChI=1S/C16H29NC	N/A	1	Positive	ECG-ACYL-AMIDES	284.222	UPDATE-SINGLE-A	Gold	NA	C16H29NO3	MTQYOVXFEYREBF	MTQYOVXFEYREBF	Lipids and lipid-like m	Fatty Acyls	Fatty amides	Fatty amides	N-acyl amines	Fatty acids	mzspec:GNPS:GNPS
same ion	O=C(NC(C(O)=O)CC	InChI=1S/C25H39NC	N/A	2	Positive	ECG-ACYL-AMIDES	418.295	UPDATE-SINGLE-A	Silver	NA	C25H39NO4	SKQHGVYVWIKEKJ	SKQHGVYVWIKEKJ	Organic acids and de	Carboxylic acids and	Amino acids, peptide	Fatty amides	N-acyl amines	Fatty acids	mzspec:GNPS:GNPS
same ion	O=C(NC(C(O)=O)C(	InChI=1S/C19H35NC	N/A	1	Positive	ECG-ACYL-AMIDES	326.269	UPDATE-SINGLE-A	Gold	NA	C19H35NO3	FIWFAXHHHSXJMC	FIWFAXHHHSXJMC	Organic acids and de	Carboxylic acids and	Amino acids, peptide	Fatty amides	N-acyl amines	Fatty acids	mzspec:GNPS:GNPS
same ion	O=C(NC(CC1=NC=C	InChI=1S/C18H29N3	N/A	2	Positive	ECG-ACYL-AMIDES	336.228	UPDATE-SINGLE-A	Silver	NA	C18H29N3O3	BWFJRCAJQSRLJY-	BWFJRCAJQSRLJY	Organic acids and de	Carboxylic acids and	Amino acids, peptide	NA	NA	Alkaloids	mzspec:GNPS:GNPS
same ion	O=C(NCCCCC)CCO	InChI=1S/C16H31NC	N/A	1	Positive	ECG-ACYL-AMIDES	254.248	UPDATE-SINGLE-A	Gold	NA	C16H31NO	UNWDDGPHGUXSL	UNWDDGPHGUXSL	Lipids and lipid-like m	Fatty Acyls	Fatty amides	Fatty amides	N-acyl amines	Fatty acids	mzspec:GNPS:GNPS
same ion	O=C(NC(C(O)=O)C(	InChI=1S/C17H31NC	N/A	1	Positive	ECG-ACYL-AMIDES	298.238	UPDATE-SINGLE-A	Gold	NA	C17H31NO3	GIBJNGPXLZPUCZ-	GIBJNGPXLZPUCZ	Organic acids and de	Carboxylic acids and	Amino acids, peptide	Fatty amides	N-acyl amines	Fatty acids	mzspec:GNPS:GNPS
same ion	O=C(NC(C(O)=O)C(	InChI=1S/C16H29NC	N/A	1	Positive	ECG-ACYL-AMIDES	300.217	UPDATE-SINGLE-A	Gold	NA	C16H29NO4	DLQMQQWJJDONN	DLQMQQWJJDONN	Organic acids and de	Carboxylic acids and	Amino acids, peptide	Fatty amides	N-acyl amines	Fatty acids	mzspec:GNPS:GNPS
same ion	O=C(NC(C(O)=O)CC	InChI=1S/C17H30N2	N/A	1	Positive	ECG-ACYL-AMIDES	327.228	UPDATE-SINGLE-A	Gold	NA	C17H30N2O4	DAGDVFYQDOYBP	DAGDVFYQDOYBP	Organic acids and de	Carboxylic acids and	Amino acids, peptide	Fatty amides	N-acyl amines	Fatty acids	mzspec:GNPS:GNPS
same ion	O=C(NCC(O)=O)CC	InChI=1S/C16H29NC	N/A	1	Positive	ECG-ACYL-AMIDES	284.222	UPDATE-SINGLE-A	Gold	NA	C16H29NO3	XBPUCXAVBXOKOL	XBPUCXAVBXOKOL	Organic acids and de	Carboxylic acids and	Amino acids, peptide	Fatty amides	N-acyl amines	Fatty acids	mzspec:GNPS:GNPS
same ion	O=C(NC(C(O)=O)C(	InChI=1S/C15H27NC	N/A	1	Positive	ECG-ACYL-AMIDES	270.206	UPDATE-SINGLE-A	Gold	NA	C15H27NO3	LBTVPCNUFWRAPL	LBTVPCNUFWRAPL	Organic acids and de	Carboxylic acids and	Amino acids, peptide	Fatty amides	N-acyl amines	Fatty acids	mzspec:GNPS:GNPS
same ion	O=C(NC(C(O)=O)CC	InChI=1S/C19H35NC	N/A	1	Positive	ECG-ACYL-AMIDES	342.264	UPDATE-SINGLE-A	Gold	NA	C19H35NO4	UISJHGBYJQEQBK-	UISJHGBYJQEQBK	Organic acids and de	Carboxylic acids and	Amino acids, peptide	Fatty amides	N-acyl amines	Fatty acids	mzspec:GNPS:GNPS
same ion	O=C(NC(CCSC)C(O	InChI=1S/C17H31NC	N/A	1	Positive	ECG-ACYL-AMIDES	330.21	UPDATE-SINGLE-A	Gold	NA	C17H31NO3S	KIODMVCNZYIFQE-	KIODMVCNZYIFQE	Organic acids and de	Carboxylic acids and	Amino acids, peptide	Fatty amides	N-acyl amines	Fatty acids	mzspec:GNPS:GNPS
same ion	O=C(NCC(O)=O)CC	InChI=1S/C14H25NC	N/A	1	Positive	ECG-ACYL-AMIDES	256.191	UPDATE-SINGLE-A	Gold	NA	C14H25NO3	FYJSGDZVYSZEGA	FYJSGDZVYSZEGA	Organic acids and de	Carboxylic acids and	Amino acids, peptide	Fatty amides	N-acyl amines	Fatty acids	mzspec:GNPS:GNPS
same ion	O=C(NCCCCN)CCO	InChI=1S/C16H32N2	N/A	1	Positive	ECG-ACYL-AMIDES	269.259	UPDATE-SINGLE-A	Gold	NA	C16H32N2O	WBPQAZOTRLKUM	WBPQAZOTRLKUM	Lipids and lipid-like m	Fatty Acyls	Fatty amides	Fatty amides	N-acyl amines	Fatty acids	mzspec:GNPS:GNPS
same ion	O=C(NC(C(O)=O)CC	InChI=1S/C16H28N2	N/A	1	Positive	ECG-ACYL-AMIDES	313.212	UPDATE-SINGLE-A	Gold	NA	C16H28N2O4	XPJHOVGAKANAH	XPJHOVGAKANAH	Organic acids and de	Carboxylic acids and	Amino acids, peptide	Fatty amides	N-acyl amines	Fatty acids	mzspec:GNPS:GNPS
same ion	O=C(NC(C(O)=O)CC	InChI=1S/C22H41N3	N/A	1	Positive	ECG-ACYL-AMIDES	412.317	UPDATE-SINGLE-A	Gold	NA	C22H41N3O4	HLGPQXBQDRNSH	HLGPQXBQDRNSH	Organic acids and de	Carboxylic acids and	Amino acids, peptide	Fatty amides	N-acyl amines	Fatty acids	mzspec:GNPS:GNPS
same ion	CCCCCCCCCCCCC	InChI=1S/C23H47NC	N/A	1	Positive	ECG-ACYL-AMIDES	392.329	UPDATE-SINGLE-A	Gold	NA	C23H47NO	BNMNWSJQGWRN4	BNMNWSJQGWRN4	Lipids and lipid-like m	Fatty Acyls	Fatty amides	Fatty amides	N-acyl amines	Fatty acids	mzspec:GNPS:GNPS
structural isomer	O=C(O)C(NC(CC(O)	InChI=1S/C16H31NC	NA	3	Positive	GNPS-LIBRARY	318.228	UPDATE-SINGLE-A	Bronze	NA	C16H31NO5	MYAXJEBANUBRM	MYAXJEBANUBRM	Organic acids and de	Carboxylic acids and	Amino acids, peptide	Fatty amides	N-acyl amines	Fatty acids	mzspec:GNPS:GNPS
structural isomer	NA	NA	NA	3	Positive	GNPS-LIBRARY	290.196	UPDATE-SINGLE-A	Bronze	NA	N/A	N/A	N/A	N/A	N/A	N/A	N/A	N/A	N/A	mzspec:GNPS:GNPS
structural isomer	O=C(O)C(NC(CC(O)	InChI=1S/C18H35NC	NA	3	Positive	GNPS-LIBRARY	346.259	UPDATE-SINGLE-A	Bronze	NA	C18H35NO5	YKQKOGLJZIAXBT-I	YKQKOGLJZIAXBT	Organic acids and de	Carboxylic acids and	Amino acids, peptide	Fatty amides	N-acyl amines	Fatty acids	mzspec:GNPS:GNPS
structural isomer	O=C(O)C(NC(CC(O)	InChI=1S/C18H35NC	NA	3	Positive	GNPS-LIBRARY	346.259	UPDATE-SINGLE-A	Bronze	NA	C18H35NO5	YKQKOGLJZIAXBT-I	YKQKOGLJZIAXBT	Organic acids and de	Carboxylic acids and	Amino acids, peptide	Fatty amides	N-acyl amines	Fatty acids	mzspec:GNPS:GNPS
structural isomer	O=C(CCCCCCCCC	InChI=1S/C20H30N2	N/A	1	Positive	GNPS-ION-MOBILIT	347.237	UPDATE-SINGLE-A	Gold	NA	C20H30N2O3	RNZCWGFBWDHKP	RNZCWGFBWDHKP	N/A	N/A	N/A	NA	NA	Alkaloids	mzspec:GNPS:GNPS
structural isomer	NA	NA	NA	3	Positive	GNPS-LIBRARY	300.217	UPDATE-SINGLE-A	Bronze	NA	N/A	N/A	N/A	N/A	N/A	N/A	N/A	N/A	N/A	mzspec:GNPS:GNPS
structural isomer	O=C(O)C(NC(CC(O)	InChI=1S/C8H15NO5	NA	3	Positive	GNPS-LIBRARY	206.102	UPDATE-SINGLE-A	Bronze	NA	C8H15NO5	MQANMGTXMZAGO	MQANMGTXMZAGO	Organic acids and de	Carboxylic acids and	Amino acids, peptide	Small peptides	Dipeptides	Amino acids and Pep	mzspec:GNPS:GNPS
structural isomer	COc1cc(CCNC(=O)C	InChI=1S/C19H31NC	N/A	1	Positive	GNPS-ION-MOBILIT	338.237	UPDATE-SINGLE-A	Gold	NA	C19H31NO4	LLXYODGHNWKABI	LLXYODGHNWKABI	N/A	N/A	N/A	Pseudoalkaloids	Capsaicins and Caps	Alkaloids	mzspec:GNPS:GNPS
structural isomer	O=C(NCCc1c[nH]c2c	InChI=1S/C15H18N2	N/A	1	Positive	GNPS-ION-MOBILIT	243.15	UPDATE-SINGLE-A	Gold	NA	C15H18N2O	XHRBVPGBQAVBG	XHRBVPGBQAVBG	Organoheterocyclic c	Indoles and derivative	Indoles	Tryptophan alkaloids	Simple indole alkaloic	Alkaloids	mzspec:GNPS:GNPS

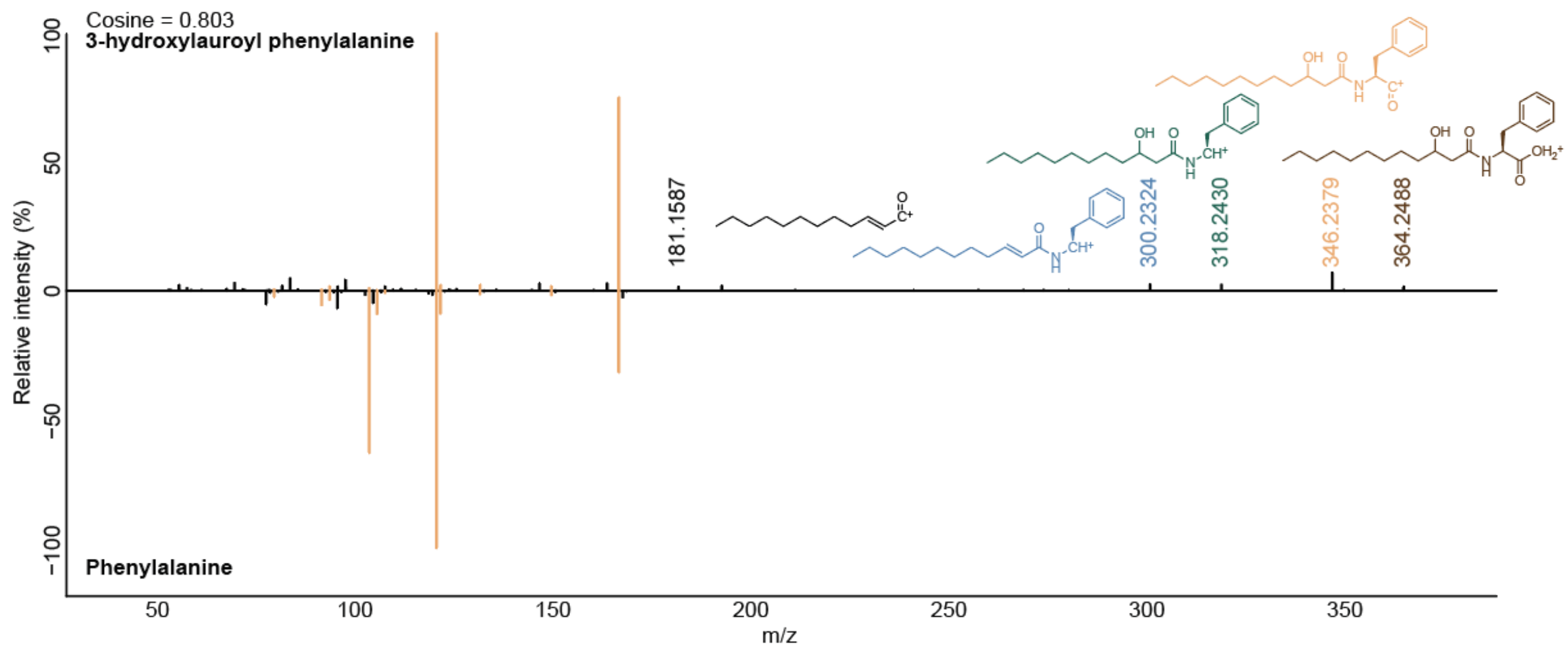

Fig. S1. Fragmentation of 3-hydroxylauroyl phenylalanine. MS/MS spectral match between 3-hydroxylauroyl phenylalanine and phenylalanine give a cosine of 0.803 because 77.7% of fragment intensity in the *N*-acyl amide originates from a peak shared with the head group. Peaks which are orange are shared between spectra. Six peaks in the top spectrum are labeled with putative structural identification that include the 3-hydroxy fatty acyl moiety.
