# Supplementary material for: Synthesis-driven reverse metabolomics reveals 3-hydroxy N-acyl amides as gut microbial molecules
Source: mSystems. 2026 Sep 24;11(10):e00839-26. doi: 10.1128/msystems.00839-26 (PMC13712783; doi:10.1128/msystems.00839-26)
Supplement: Supplementary file 2 [file reviewer-comments.pdf]

ASM | mSystems

Synthesis-driven reverse metabolomics reveals 3-hydroxy N-acyl amides as gut microbial molecules

Victoria Deleray, Vincent Charron-Lamoureux, Kyle Vittali, Helena Mannochio-Russo, Crystal Wang, Corinn Walker, Karsten Zengler, Dionicio Siegel, and Pieter Dorrestein

Corresponding Author(s): Pieter Dorrestein, University of California, San Diego

Review Timeline:

Submission Date:	June 30, 2026
Editorial Decision:	July 16, 2026
Revision Received:	August 9, 2026
Accepted:	August 13, 2026

Editor: Christopher Anderton

Reviewer(s): Disclosure of reviewer identity is with reference to reviewer comments included in decision letter(s). The following individuals involved in review of your submission have agreed to reveal their identity: Melanie Odenkirk (Reviewer #3)

Transaction Report:

(Note: With the exception of the correction of typographical or spelling errors that could be a source of ambiguity, letters and reports are not edited. The original formatting of letters and referee reports may not be reflected in this compilation.)

DOI: https://doi.org/10.1128/msystems.00839-26

Re: mSystems00839-26 (Synthesis-driven reverse metabolomics reveals 3-hydroxy N-acyl amides as gut microbial molecules)

Dear Dr. Pieter C. Dorrestein:

Thank you for the privilege of reviewing your work. Below you will find my comments, instructions from the mSystems editorial office, and the reviewer comments.

Please return the manuscript within 60 days; if you cannot complete the modification within this time period, please contact me. If you do not wish to modify the manuscript and prefer to submit it to another journal, notify me immediately so that the manuscript may be formally withdrawn from consideration by mSystems.

Revision Guidelines
To submit your modified manuscript, log into the submission site at https://msystems.msubmit.net/cgi-bin/main.plex. Go to Author Tasks and click the appropriate manuscript title to begin. The information you entered when you first submitted the paper will be displayed; update this as necessary. Note the following requirements:

• Upload point-by-point responses to the issues raised by the reviewers in a file named "Response to Reviewers," NOT in your cover letter.
• Upload a compare copy of the manuscript (without figures) as a "Marked-Up Manuscript" file.
• Upload a clean .DOC/.DOCX version of the revised manuscript and remove the previous version.
• Each figure must be uploaded as a separate, editable, high-resolution file (TIFF or EPS preferred), and any multipanel figures must be assembled into one file.
• Any supplemental material intended for posting by ASM should be uploaded with their legends separate from the main manuscript. You can combine all supplemental material into one file (preferred) or split it into a maximum of 10 files with all associated legends included.

For complete guidelines on revision requirements, see our Submission and Review Process webpage. Submission of a paper that does not conform to guidelines may delay acceptance of your manuscript.

Data availability: ASM policy requires that data be available to the public upon online posting of the article, so please verify all links to sequence records, if present, and make sure that each number retrieves the full record of the data. If a new accession number is not linked or a link is broken, provide mSystems production staff with the correct URL for the record. If the accession numbers for new data are not publicly accessible before the expected online posting of the article, publication may be delayed; please contact production staff (mSystems@asmusa.org) immediately with the expected release date.

Publication Fees: For information on publication fees and which article types are subject to charges, visit our website. If your manuscript is accepted for publication and any fees apply, you will be contacted separately about payment during the production process; please follow the instructions in that e-mail. Arrangements for payment must be made before your article is published.

ASM Membership: Corresponding authors may join or renew ASM membership to obtain discounts on publication fees. Need to upgrade your membership level? Please contact Customer Service at Service@asmusa.org.

The ASM Journals program strives for constant improvement in our submission and publication process. Please tell us how we can improve your experience by taking this quick Author Survey.

Thank you for submitting your paper to mSystems.

Sincerely,
Christopher Anderton
Editor
mSystems

Reviewer #3 (Comments for the Author):

Please see the attached document for comments.

Review of

Synthesis-driven reverse metabolomics reveals 3-hydroxy N-acyl amides as gut microbial molecules

In this manuscript, Deleray et al. present a comprehensive investigation into an under-represented class of small molecules called 3-hydroxy N-acyl amides through an innovative reverse synthesis approach previously adopted for bile acid conjugates and other chemical structures. Overall, the manuscript is very well written, including a thoughtful scientific rationale and well-curated figures to demonstrate evidence for 3-hydroxy N-acyl amides. The authors have satisfactorily addressed most of the reviewers' previous comments, further strengthening the manuscript. The remaining comments reflect additional clarifications for method transparency and library adoption.

1. It seems prudent to emphasize why 3-hydroxy N-acyl amides are worth prioritizing for the reverse metabolomics study performed here with only 0.026% of the unannotated spectra following lines 133-136 in the results and discussion. The biological evidence for these molecules is well-stated in the introduction but could be reiterated here to further justify the forthcoming results.

2. In performing spectra matching within the public domain, the authors note matches with cosine similarity scores > 0.7 for 161,626 spectra. Since MS/MS fragmentation varies across instrumentation, have the authors noticed a bias in these public domain matches? This seems important to consider for the utility of the reference library across laboratories where instrumentation may differ from that used to generate this library.

3. Line 298 includes author changes that should be revised.

4. The LC methodologies still appear to contain discrepancies. In the *Curation of the 3-hydroxy N-acyl amide library* section, the separation reads "The gradient is 0-0.5 min, 5% B; 0.5-7.5 min, 5-40% B; 7.5-9 min, 40-99% B; 9-10 min, 99%B; 10.1-12 min, 5%B." while in section *Retention time and drift time analysis* the separation reads "Samples were eluted at 0.5 mL/min using the following gradient: 0-0.5 min, 5% B; 1.1 min, 25% B; 7.5 min, 40% B; 8.5 min, 99% B; 10.0 min, 99% B; 10.1 min, 5% B; 12.0 min,5% B." The second method appears to include a step at the 1.1 min mark that is absent from the first method. Similarly, the hold for the second method at 99% B begins 0.5 minutes before the hold in the first method.

5. Response to the comment regarding the low signal for the example case of 3-hydroxytetradecanoyl glutamic acid appear to have removed the raw intensity value

altogether. It seems more favorable to be transparent on the low signal as this could impact biological claims.

Response to Reviewers

Revision of: Synthesis-driven reverse metabolomics reveals 3-hydroxy *N*-acyl amides as gut microbial molecules

We thank the Reviewers and Editorial team for the second assessment of our manuscript, and appreciate the opportunity to resubmit. We have addressed each of these comments (black) comprehensively in a point-by-point manner. Changes in the original manuscript text will be highlighted in yellow in the modified text. The modified text and location are provided within this response letter (in green and highlighted in yellow) for convenience. Text from the manuscript that has not been changed is included in green for reference.

Reviewer comment: In this manuscript, Deleray et al. present a comprehensive investigation into an under- represented class of small molecules called 3-hydroxy N-acyl amides through an innovative reverse synthesis approach previously adopted for bile acid conjugates and other chemical structures. Overall, the manuscript is very well written, including a thoughtful scientific rationale and well-curated figures to demonstrate evidence for 3-hydroxy N-acyl amides. The authors have satisfactorily addressed most of the reviewers' previous comments, further strengthening the manuscript. The remaining comments reflect additional clarifications for method transparency and library adoption.

Response: We thank the Reviewer for the thoughtful and comprehensive review of our manuscript, and are pleased that most comments have been satisfactorily addressed.

Reviewer comment 1. It seems prudent to emphasize why 3-hydroxy N-acyl amides are worth prioritizing for the reverse metabolomics study performed here with only 0.026% of the unannotated spectra following lines 133-136 in the results and discussion. The biological evidence for these molecules is well-stated in the introduction but could be reiterated here to further justify the forthcoming results.

Response: We appreciate this comment and agree further context is needed. This percentage could only be calculated after the creation of the library, and only includes 3-hydroxy *N*-acyl amides that were included in this reference database, but fails to include other conjugations of different amines and lipid chains, which potentially exist but remain unannotated. Rather than presenting these results too early, we have now rephrased the justification.
Lines 131-139 now reads:

"At present, the average annotation rate of all MS/MS using publicly accessible reference libraries in the major repositories Metabolights, Metabolomics Workbench, and GNPS/MassIVE is roughly 8%(7). This annotation gap is particularly limited for metabolite classes such as 3-hydroxy *N*-acyl amides, for which a few individually characterized molecules have demonstrated important biological roles, but no systemic reference spectral library exists. Their modular composition of diverse 3-hydroxy fatty acid tails conjugated to various amines suggests that the few reported structures represent only a fraction of the chemical diversity that exists for these molecules. To

facilitate their annotation, we aimed to create a reference library by performing 55 multiplexed reactions containing mixtures of 19 3-hydroxy fatty acids, and 42 amine head groups **(Table S1)**.**"**

Reviewer comment 2. In performing spectra matching within the public domain, the authors note matches with cosine similarity scores > 0.7 for 161,626 spectra. Since MS/MS fragmentation varies across instrumentation, have the authors noticed a bias in these public domain matches? This seems important to consider for the utility of the reference library across laboratories where instrumentation may differ from that used to generate this library.

Response: We agree with the Reviewer that fragmentation patterns of an ion differ between types of instrumentation used, ToF versus Orbitrap for example. In the metadata available on Pan-ReDU, samples may include the mass spectrometer used for acquisition. Among the metadata, 16 different instruments produced an MS/MS that had a match to the 3-hydroxy *N*-acyl amide library. These can be characterized between ToF and Orbitrap instruments. 15.4% of the matches are labeled to an Orbitrap and 1.7% of the matches are labeled to a ToF. The data used for this calculation has already been deposited in Github.

Lines 336-346 now read:
"Similarly, with all the different instrumentation and analytical methods that exist for LC-MS/MS workflows(23), it is likely that the pan-repository search was not comprehensive of all 3-hydroxy *N*-acyl amides in the library, and the results are likely biased to similar methods used in the creation of the library. As such, results are likely to change with the increase of data deposition in the repositories used here. We recognize that obtaining spectra from multiple instruments or analytical methods, such as negative ionization mode, would improve the library and serve as a future direction. For example, based on available metadata shared on the instrumentation type used in the FASST search, 15.4% of MS/MS matches are to an Orbitrap instrument, whereas 1.7% to ToF, which may be explained by and highlight that the library which was created on an Orbitrap instrument can be more readily adopted by experimental methods also utilizing an Orbitrap.**"**

Reviewer comment 3. Line 298 includes author changes that should be revised.

Response: We thank the Reviewer for catching this mistake.

Lines 300-303 now read:

"We found that this class of 3-hydroxy *N*-acyl amides is found in hundreds of LC-MS/MS studies among diverse types of organisms and human samples and we demonstrated that community cultures of gut bacteria, when given 3-hydroxy lipids as substrates, can lead to the production of *N*-acyl amides.**"**

Reviewer comment 4. The LC methodologies still appear to contain discrepancies. In the *Curation of the 3- hydroxy N-acyl amide library* section, the separation reads "The gradient is 0-0.5 min, 5% B; 0.5-7.5 min, 5-40% B; 7.5-9 min, 40-99% B; 9-10 min, 99%B; 10.1-12 min,

5%B.” while in section *Retention time and drift time analysis* the separation reads “Samples were eluted at 0.5 mL/min using the following gradient: 0-0.5 min, 5% B; 1.1 min, 25% B; 7.5 min, 40% B; 8.5 min, 99% B; 10.0 min, 99% B; 10.1 min, 5% B; 12.0 min,5% B.” The second method appears to include a step at the 1.1 min mark that is absent from the first method. Similarly, the hold for the second method at 99% B begins 0.5 minutes before the hold in the first method.

Response: We thank the Reviewer for re-addressing this comment. Although retention times were evaluated only within the second method and were not compared directly between the two gradients, we have looked at the raw files to now report the original method used.

Lines 375-376 now read:

“The gradient is 0-0.5 min, 5% B; 1.1 min, 25% B; 7.5 min, 40% B; 8.5 min, 99% B; 10.0 min, 99% B; 10.1 min, 5% B; 12.0 min, 5% B.**”**

Reviewer comment 5. Response to the comment regarding the low signal for the example case of 3- hydroxytetradecanoyl glutamic acid appear to have removed the raw intensity value altogether. It seems more favorable to be transparent on the low signal as this could impact biological claims.

Response: To address the Reviewer comment, we have re-added information on the metabolite detection in soil, but have cautioned the reader of its limited identification.

Lines 278-283 now read:

“Reanalysis of a dataset which sampled human cadavers’ skin showed the abundance of 3-hydroxytetradecanoyl glutamic acid significantly increased (p < 0.05) with advancement of skin decomposition (**Fig. 4e**). This metabolite was detected in the soil surrounding the cadavers with MS1, RT, and DT, however its low peak intensity (<150) did not trigger MS/MS, limiting confidence of its identification and therefore the full interpretation of its presence in the soil.**”**

Re: mSystems00839-26R1 (Synthesis-driven reverse metabolomics reveals 3-hydroxy N-acyl amides as gut microbial molecules)

Dear Dr. Pieter C. Dorrestein:

Your manuscript has been accepted, and I am forwarding it to the ASM production staff for publication. Your paper will first be checked to make sure all elements meet the technical requirements. ASM staff will contact you if anything needs to be revised before copyediting and production can begin. Otherwise, you will be notified when your proofs are ready to be viewed.

Data Availability: ASM policy requires that data be available to the public upon online posting of the article, so please verify all links to sequence records, if present, and make sure that each number retrieves the full record of the data. If a new accession number is not linked or a link is broken, provide production staff with the correct URL for the record. If the accession numbers for new data are not publicly accessible before the expected online posting of the article, publication may be delayed; please contact ASM production staff immediately with the expected release date.

Publication Fees: For information on publication fees and which article types have charges, please visit our website. We have partnered with Copyright Clearance Center (CCC) to collect author charges. If fees apply to your paper, you will receive a message from no-reply@copyright.com with further instructions. For questions related to paying charges through RightsLink, please contact CCC at ASM_Support@copyright.com or toll free at +1-877-622-5543. CCC makes every attempt to respond to all emails within 24 hours.

ASM Membership: Corresponding authors may join or renew ASM membership to obtain discounts on publication fees. Need to upgrade your membership level? Please contact Customer Service at Service@asmusa.org.

Embargo Policy: A press release may be issued as soon as the manuscript is posted on the mSystems Latest Articles webpage. The corresponding author will receive an email with the subject line "ASM Journals Author Services Notification" when the article is available online.

Cover Image Submissions: If you would like to submit a potential Cover Image, please email a file and a short legend to msystems@asmusa.org. Please note that we can only consider images that (i) the authors created or own and (ii) have not been previously published. By submitting, you agree that the image can be used under the same terms as the published article. Image File requirements: TIF/EPS, 7.5 inches wide by 8.25 inches tall (at least 2,250 pixels wide by 2,475 pixels tall), minimum 300 dpi resolution (600 dpi preferred), RGB, and no figure elements, e.g., arrows or panel labels. The legend should be a short description of the image, 1-2 sentences recommended. Please use the ASM Journals Cover Image Sizing Tool (https://journals.asm.org/pb-assets/html/asm-cover-sizing-tool.html) to ensure that your proposed cover image meets our size requirements.

Thank you for submitting your paper to mSystems.

Sincerely,
Christopher Anderton
Editor
mSystems
